# Generalizable Direct Protein Sequencing With InstaNexus

**DOI:** 10.1016/j.mcpro.2026.101547

**Published:** 2026-03-02

**Authors:** Marco Reverenna, Maike Wennekers Nielsen, Darian Stephan Wolff, Jemma Daniel, Elpida Lytra, Suthimon Thumtecho, Pasquale D. Colaianni, Anne Ljungars, Andreas H. Laustsen, Erwin M. Schoof, Jeroen Van Goey, Timothy P. Jenkins, Marie V. Lukassen, Alberto Santos, Konstantinos Kalogeropoulos

**Affiliations:** 1 Novo Nordisk Foundation Center for Biosustainability, Technical University of Denmark, Lyngby, Denmark; 2 Department of Biotechnology and Biomedicine, Technical University of Denmark, Lyngby, Denmark; 3 Novonesis, Lyngby, Denmark; 4 InstaDeep Ltd, London, UK; 5 Center for Translational Protein Design, Technical University of Denmark, Lyngby, Denmark; 6 Department of Bionanoscience, Delft University of Technology, Delft, Netherlands; 7 Kavli Institute of Nanoscience, Delft, Netherlands

**Keywords:** de novo sequencing, protein sequencing, protein assembly, therapeutics, sample preparation

## Abstract

Accurate determination of protein sequences is central to biology. Protein-based therapeutics, such as antibodies and nanobodies, are not encoded in reference genomes, challenging their accurate characterization via standard proteomics. Current methods rely on indirect inference, fragmented outputs, and labor-intensive workflows, which hinder functional insights and routine application. Here, we present a generalizable, end-to-end workflow for direct protein sequencing, combining streamlined sample preparation, artificial intelligence (AI)-driven *de novo* peptide sequencing, and tailored assembly to reconstruct contiguous protein sequences. A novel composite scoring framework prioritizes longer assemblies and coverage, enhancing accuracy and reducing ambiguity. Validation across diverse protein modalities demonstrates its utility and ability to robustly sequence functionally critical regions of selected proteins. This workflow represents an advance in precision proteomics with promising applications in therapeutic discovery, immune profiling, and protein science.

## Highlights

• End-to-end workflow for reference-free direct protein sequencing.
• Multiprotease digestion optimized for de novo peptide assembly.
• AI-driven de novo sequencing with FDR control improves assembly accuracy.
• Composite scoring prioritizes workflow optimization for accurate protein assemblies.
• Robust sequencing of antibodies, nanobodies, and designed binders.

## Graphical Abstract

**Figure:**
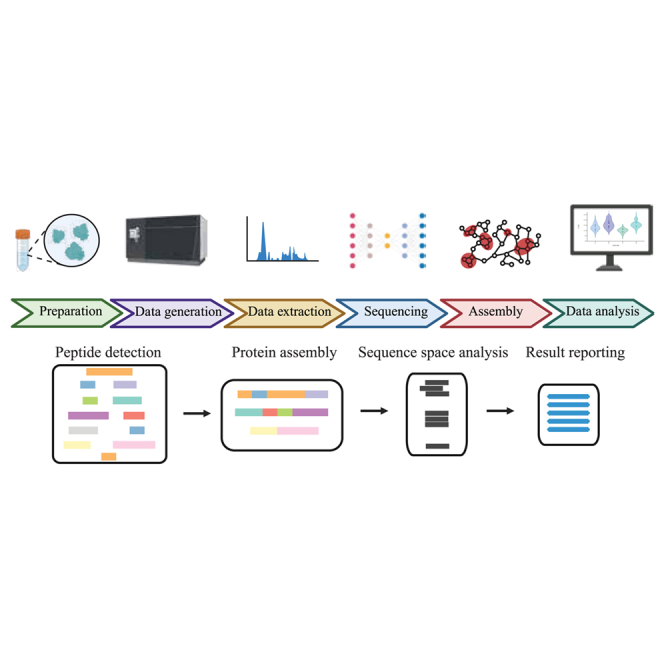

## In Brief

Direct protein sequencing remains challenging for proteins lacking genomic references. Here, we present InstaNexus, a generalizable end-to-end workflow that integrates optimized multiprotease digestion, AI-driven de novo peptide sequencing with false discovery rate control, and tailored assembly strategies. By prioritizing accurate and concise assemblies, InstaNexus enables robust reconstruction of functionally critical protein regions from low-input samples, facilitating practical applications in therapeutic discovery, immune profiling, and protein science.

Accurate protein sequencing remains a fundamental challenge in biology, with the potential to unlock a wide range of applications, from the discovery of novel enzymes and therapeutic proteins to insights into evolutionary relationships and disease mechanisms (1, 2, 3, 4, 5, 6). This is particularly relevant for complex protein classes such as antibodies, nanobodies, and increasingly, *de novo* designed binders, which play indispensable roles in diagnostics, therapeutics, and biomedical research (7, 8, 9, 10, 11, 12, 13). Their high specificity and affinity for target antigens enable applications ranging from cancer therapies (14) to viral neutralization (15) and biomarker discovery (16). Many antibodies are still generated through animal immunization or selected from (polyclonal) antibody libraries, where it is crucial to have a direct link between their genotype and phenotype (e.g., using B-cell sequencing or display technologies) (17, 18, 19) to obtain their accurate sequence if the antibodies are to be recombinantly expressed (20). Often, this entails cumbersome processes of handling living cells from animals or human donors, constructing antibody libraries, and/or access to costly equipment and highly trained scientists, which creates large barriers for many scientists that wish to sequence their antibodies, especially in the reagents field (17). In addition, antibody sequencing can be pivotal for mapping immune responses, guiding vaccine development, and supporting precision medicine approaches requiring detailed immune profiling (21, 22, 23).

In addition to antibodies, reliable protein sequencing can find utility for sequencing of novel or engineered proteins for biothreat detection and biosafety, as advancements in generative protein design technology heighten potential risks associated with engineered proteins (24, 25). Rapid sequencing of proteins would enable early detection and intervention, allowing safety measures to be put in place faster. Beyond these applications, direct protein sequencing also holds promise in broader biomedical research, such as biomarker and enzyme discovery (26, 27) and mapping of protein-protein interaction networks (28).

In contrast to nucleic acids, proteins cannot be readily amplified by polymerase chain reaction (PCR). As a result, while trace amounts of DNA or RNA are sufficient for high-throughput sequencing, protein sequencing requires significantly larger sample sizes. Currently, bottom-up mass spectrometry (MS), involving proteolytic digestion followed by liquid chromatography-tandem mass spectrometry (LC-MS/MS) (29), is commonly employed for protein sequencing (4, 30, 31). However, these approaches depend heavily on existing reference sequences from a proteome (32, 33), limiting their utility for highly variable regions, like the complementarity-determining regions (CDRs) of antibodies, and are not applicable to novel proteins.

To address these limitations, strategies such as open searches and multiprotease digestion have been introduced to increase flexibility and sequence coverage. Open searches in proteomics allow identification of peptides without predefined posttranslational modifications (34) or protease cleavage specificity (35, 36), but the broad search space introduces significant false discovery rate (FDR) control challenges, increased computational costs, and is still dependent on reference databases. Similarly, utilizing multiple proteases for peptide generation enhances sequence coverage but complicates sample preparation and downstream analyses due to diverse cleavage specificities and increased methodological complexity (37, 38).

To circumvent these limitations, *de novo* peptide sequencing, which operates independently of reference sequence data, has emerged as a promising approach. Recent computational advancements, primarily through introduction of deep learning models, have significantly improved accuracy (39, 40, 41). Nevertheless, critical challenges persist, including false-positive identifications, limited diversity in enzymatic digestion, sensitivity of assembly algorithms to sequencing errors, and the absence of robust workflows for peptide assembly into contiguous protein sequences.

In this study, we address the challenges associated with protein sequencing using mass spectrometry by introducing an enhanced methodology integrating optimized sample preparation protocols and advanced computational techniques enabling improved accuracy in protein sequence determination. We use a complementary multienzyme digestion strategy that increases peptide diversity and generates overlapping ends, while maintaining simple and fast sample preparation. We utilize artificial intelligence-driven *de novo* peptide sequencing with rigorous post processing and filtering of predicted peptides, reducing the number of false positives and facilitating accurate protein sequence assembly. Finally, we implement a protein sequence assembly method optimized to different protein modalities, along with alignment and clustering steps to refine and prioritize unique candidate sequences that span contiguous regions of target proteins. This integrated approach establishes a robust, template-agnostic platform for direct protein sequencing, with significant implications for the discovery of biomarkers, novel enzymes and biologics, as well as biothreat detection. Our direct protein sequencing workflow can be used to sequence any protein given enough purity, further expanding its applicability across diverse biological and industrial applications.

## Experimental Procedures

### Samples

We used four different sample types to develop, optimize, and evaluate our methodology. These ranged in complexity and biological origin. Recombinant bovine serum albumin (BSA) was obtained by Sigma-Aldrich (cat no. A2153).

The nanobodies (hereafter named Nb1-10) included in this study were discovered using phage display technology as described previously (42). A list of the nanobodies alongside the methods used for their expression and purification has been described previously (41).

The antibodies (hereafter named mAbB1-mAb5) included in this study are a WT human immunoglobulin (hIg) G1 antibody (2554_01_D11) (43), three hIgG1 antibodies with LALA (44) and YTE (45) mutations (TPL0179_01_C08 (46), TPL0552_02_A05 (47), and TPL0039_05_A03) (47), and an hIgG1 with LALA mutation (2555_01_A01) (43). All mAbs were discovered using phage display technology, reformatted from an scFv to a full-length IgG format and expressed in CHO cells followed by protein A purification as described previously (43).

The three *de novo* mini-binders (miBds) (NY1-B04, SILSY1-G05, and SILSY1-B11) included in this study (hereafter named miBd1-3) were designed to bind the shared cancer antigens presented on the peptide major histocompatibility complex (pMHC). A detailed outline of the design process, as well as biophysical and functional characterization has been published previously (48). In brief, NY1-B04 was designed and identified to bind the peptide major histocompatibility complex HLA-A∗02:01 presenting the peptide ‘SLLMWITQC’, while SILSY1-G05 and SILSY1-B11 are capable of binding to the neo-cancer antigen peptide ‘RVTDESILSY’ presented on HLA-A∗01:01.

The miBds have been expressed in *Bacillus subtilis* A164Δ5 derivative (48) and purified employing 6xHis-tag affinity purification. Briefly, the amino acid sequence of the miBd designs were complimented with an N-terminal secretion signal peptide from Savinase (MKKPLGKIVASTALLISVAFSSSIASA) and a C-terminal linking region (‘GGGGSEAAAKGGGGS’), in the case SILSY1-G05 and SILSY1-B11, followed by an Avitag (‘GLNDIFEAQKIEWHE’) and a 6xHis-tag (‘HHHHHH’). For NY1-B04, the linking region was omitted. The miBds were ordered as synthetic gene fragments and cloned in the expression vector pDG268Δneo. A recombinant clone containing the integrated expression construct was identified and grown in liquid culture medium.

For miBd purification, 250 ml of the sample containing cultures were subjected to centrifugation (10,000*g* for 15 min, Sorvall RC 6 plus, 46915 Thermo Fisher Scientific), and the supernatant applied to single-use columns for immobilized metal affinity chromatography (His GraviTrap columns, 11003399, Cytiva) following the supplier’s protocol. Subsequently, the samples were desalted employing disposable PD-10 columns packed with Sephadex G-25 resin (17085101, Cytiva) and eluted in 5 ml of buffer solution (50 mM Tris, 150 mM NaCl, pH 7.4). Sample accession codes are provided in Supplementary Table S1.

### Sample Preparation for Mass Spectrometry

#### Bovine Serum Albumin

The digestion protocol was optimized using BSA obtained in lyophilized form. BSA (molecular weight 66.000 g/mol) was reconstituted in a sodium deoxycholate (SDC) lysis buffer containing 1% SDC, 200 mM Tris–HCl (pH 8.5), 10 mM Tris(2-carboxyethyl) phosphine hydrochloride (TCEP), and 40 mM 2-chloroacetamide (CAA). For each enzyme and condition, 10 μg of protein was transferred to Eppendorf Protein LoBind tubes and heated at 95 °C for 5 min to ensure complete denaturation. Samples were then diluted 1:1 with 100 mM ammonium bicarbonate (AMBIC, pH 8.5) prior to enzyme addition. We tested a range of enzyme-to-protein ratios (1:50, 1:100, and 1:200), incubation times (1 h, 4 h, and 18 h), and temperatures (23 °C and 37 °C) for the following proteases: papain (Sigma-Aldrich, P5306-25MG), proteinase K (Promega, V3021), chymotrypsin (Promega, V1061), GluC (Promega, V1651), thermolysin (Promega, P1512), and elastase (Sigma-Aldrich, 324681-50UG). Thermolysin was tested at 37 °C and 70 °C, and not at 23 °C. For thermolysin, chymotrypsin, and proteinase K, CaCl_2_ was added to a final concentration of 10 mM to act as a cofactor. Trypsin (Promega, V5280) and LysC (Wako, 125-05061) were only tested at 1:50 and 1:100 enzyme-to-protein ratio at 37 °C at the three incubation times.

Digestion was quenched by a 1:1 dilution with 2% trifluoroacetic acid (TFA), after which the samples were centrifuged at 14,000*g* for 20 min to pellet the precipitated SDC. The resulting supernatants were desalted using SOLAμ SPE plates (Thermo Fisher Scientific, 60209-001), with all steps driven by centrifugation at 350*g*. The filters were activated with 200 μl of 100% methanol followed by 200 μl of 80% acetonitrile containing 0.1% formic acid. Columns were equilibrated twice with 200 μl of 1% TFA in 3% acetonitrile before sample loading. After loading, peptides were washed twice with 0.1% formic acid and subsequently eluted into clean 0.5 ml Eppendorf tubes using 40% acetonitrile with 0.1% formic acid. Eluates were concentrated in a SpeedVac (Eppendorf) and reconstituted in 12 μl of buffer A∗ (2% acetonitrile, 1% TFA) for peptide quantification by NanoDrop (DeNovix).

In addition to the enzymes with optimal activity at neutral pH, we also tested the performance of pepsin (Promega, V1959), legumain (Jena Bioscience, PR-967S), and Krakatoa (CinderBio, CB23726), which require acidic conditions. For these, BSA was reconstituted in a buffer containing 20 mM potassium phosphate and 20 mM citric acid, adjusted to pH 3 for pepsin and Krakatoa, and to pH 5.5 for legumain. TCEP and CAA were added to final concentrations of 10 mM and 40 mM, respectively, and the protein was denatured by heating to 95 °C for 5 min. Krakatoa was tested at a ratio of 1U enzyme per 5 μg protein (according to the supplier’s instructions) and incubated at 80 °C for 5, 30, and 60 min. Pepsin and legumain were evaluated under the same conditions used for the neutral pH enzymes. All reactions were quenched by placing the tubes on ice, and peptide clean-up and reconstitution was performed as described above.

After reconstitution in A∗ buffer, 50 ng of each sample was analyzed on LC-MS. Data were analyzed to find conditions, which result in high numbers of peptides while simplifying sample preparation, to avoid overly complex experimental setup. The resulting conditions were used for the processing of Nbs, mAbs, and miBds.

#### Nanobodies

A total of 10 μg of each Nb for each enzyme digest was diluted with SDC lysis buffer to a final volume of 100 μl. CaCl_2_ was added to a final concentration of 10 mM to the SDC lysis buffer used for samples that were to be digested with chymotrypsin, proteinase K, or thermolysin. Samples were heated to 95 °C for 5 min and diluted 1:1 with 100 mM AMBIC, pH 8.5. Enzymes were added in the following enzyme-to-protein ratios; papain, GluC, and elastase 1:50, trypsin 1:100, and chymotrypsin, proteinase K, and thermolysin 1:200. All samples were incubated at 37 °C for 4 h, except the proteinase K digests, which were acidified after 1 h. Samples were acidified by diluting 1:1 with TFA 2%. Samples were centrifuged at 14,000*g* for 20 min to pellet the precipitated SDC. In addition, nanobodies were digested using Krakatoa (CinderBio, CB23726) and Vesuvius (CinderBio, CB14057). Here, 10 μg of the nanobodies were mixed with TCEP and the provided digestion buffer according to the supplier’s instructions. pH was confirmed to be 3 and enzyme was added in a 2U/μg sample ratio. Samples were incubated at 80 °C for 30 min and put on ice to inactivate the enzymes. Supernatants containing resulting peptides were desalted and reconstituted using the above-mentioned procedure. A total amount of 100 ng of peptides from each sample was injected and analyzed with LC-MS/MS.

#### Antibodies

A total of 10 μg of each antibody or antibody mix for each enzyme digest was diluted with SDC lysis buffer to a final volume of 100 μl. Samples were heated to 95 °C for 5 min and diluted 1:1 with 100 mM AMBIC pH 8.5. Calcium chloride was added to a final concentration of 10 mM to the mAbs for digestion with thermolysin, proteinase K, and chymotrypsin. The proteases papain, GluC, elastase, trypsin, chymotrypsin, proteinase K, and thermolysin were added to the respective samples following the same ratios, incubation times, and temperatures as mentioned in Nanobodies section. TFA was added to a final concentration of 1% to stop enzyme activity and precipitate SDC, and samples were centrifuged at 14,000*g* for 20 min. Supernatants were desalted and reconstituted following the same procedure as above. A total of 100 ng of each sample was analyzed with LC-MS/MS. For the low-input experiment, 2 μg of mAb1 was used for each enzyme digest, and 100 ng of the digests was analyzed with LC-MS/MS.

#### Mini-binders

miBds were prepared for MS analysis with the exact same protocol as nanobodies.

### MS Acquisition

Peptides were loaded for analysis onto a 2 cm C18 trap column (Thermo Fisher Scientific 164946), connected in-line to a 15 cm C18 reversed-phase analytical column (Thermo EasySpray ES904) using 100% Buffer A (0.1% formic acid in water) at 750 bar, on the Thermo EasyLC 1200 HPLC system, with the column oven set to 30 °C.

Peptides were eluted using a 35-min gradient at a flow rate of 250 nl/min. The gradient began with a transition from 10% to 23% Buffer B (80% acetonitrile and 0.1% formic acid) over 17 min and then increased to 38% Buffer B over 6 min. This was followed by ramping up to 60% Buffer B of 3 min, after which Buffer B was increased to 95% over 3 min, holding at this concentration for 6 min.

The Q-Exactive instrument (Thermo Fisher Scientific) was run in a DD-MS2 top 10 method. Full MS spectra were collected at a resolution of 70,000, with an automatic gain control (AGC) target of 3 × 10 e^6^ or a maximum injection time of 20 ms and a scan range of 300 to 1750 m/z. The MS2 spectra were obtained at a resolution of 17,500, with an AGC target value of 1 × 10 e^6^ or a maximum injection time of 60 ms, a normalized collision energy of 25, and an intensity threshold of 1.7 × 10 e^4^. Dynamic exclusion was set to 30 s, and ions with a charge state 1, >6 or unknown were excluded. MS performance was evaluated by running complex cell lysate quality control standards.

The low input digestion was analyzed on an Orbitrap Exploris 480 with high-field asymmetric waveform ion mobility spectrometry (FAIMS) Pro installed, coupled to a Vanquish Neo (Thermo Fisher Scientific) with a PepMap Neo Trap Cartridge precolumn and an Ion Optics Aurora Rapid 8 × 75 XT C18 UHPLC column. A 38-min active gradient (linear increase starting at 4% Buffer B, increasing to 10% at minute 4, 22.5% at minute 23.1, 45% at minute 31.5, and 95% at minute 32) was used, with the column heated at 50 °C and the autosampler at 7 °C. FAIMS was operated at standard resolution with carrier gas flow set to 3.6 L/min. Two compensation voltages (CV) of −50 and −70 were used to acquire scans. MS scans were acquired at 60,000 resolution, with normalized AGC set to 300%, radio frequency (RF) lens at 40%, and injection time set to automatic. Data-dependent MS/MS scans were acquired for the most abundant precursors when available, with an isolation window of 1.6 m/z, Orbitrap resolution of 15,000, first mass at 120 m/z, normalized AGC of 75%, and automatic maximum injection time in centroid mode.

### Database Search

Raw files were analyzed using Proteome Discoverer 2.4 (Thermo Fisher Scientific) software. The LC-MS/MS raw files were processed in batches based on the enzyme used for cleavage and with the use of label-free quantitation. SequestHT was used as a search engine, matching spectra against the uniprot bovine database for the BSA samples, contaminant database, and known mAb sequences. The precursor mass tolerance was set to 10 ppm, and the fragment ion mass tolerance was set to 0.02 Da. Two missed cleavages were allowed, with peptide length between 6 and 144. Cysteine carbamidomethylation (+57.021 Da) was set as fixed modification, with methionine oxidation (+15.995 Da) and protein N-terminal acetylation (+42.011 Da) and methionine loss (−131.040 Da) included as variable modifications. Percolator was used for FDR control, with strict (0.01) and relaxed (0.05) target FDR on peptide-spectrum match (PSM), peptide, and protein level. The Minora Feature Detector node was used for quantitation. Nbs and miBds were analyzed directly with *de novo* sequencing.

### *De novo* Peptide Sequencing

We performed *de novo* peptide sequencing using InstaNovo v0.1.4 (41). We first converted raw MS data into the standardized.mzML format using ProteoWizard MSConvert (49). We put the resulting .mzML files into the InstaNovo framework, which we deployed on a high-performance computing (HPC) cluster (https://www.hpc.dtu.dk/). The InstaNovo (41) predictions were subjected to rescoring and decoy-free FDR estimation using Winnow (50), a calibration model that is trained to estimate the probability of PSM correctness using MS features. Results were exported and used for protein assembly and downstream analysis.

### Protein Sequencing and Assembly

#### Data Processing

The data processing steps after *de novo* peptide inferencing include the following key steps in the order they are presented: We converted InstaNovo’s logarithmic confidence score to its exponent for improved interpretability, obtaining a confidence score from 0 to 1 for each peptide prediction. We stripped modification strings included in the peptide sequences (e.g., (ox)) to standardize formatting and enable sequence mapping. We discarded rows that contain missing values and filtered out peptides shorter than seven or longer than 20 amino acids to enhance identification reliability. Subsequently, we filtered PSMs based on their FDR control. Finally, we excluded predictions that map to known contaminants, such as albumin (with the exception of BSA) and collagen sequences.

#### Greedy Peptide Assembly

We performed peptide assembly using two complementary strategies: a greedy overlap-based method and a de Bruijn graph (DBG) approach, each designed to maximize sequence reconstruction based on the predictions generated by InstaNovo.

The greedy assembler operates by identifying complete overlaps between PSMs, specifically where the suffix of one peptide aligns with the prefix of another or where one sequence is fully contained within another. Sequences are iteratively merged based on a defined minimum overlap threshold, applying a “longest-overlap-first” heuristic to prioritize the most significant alignments. To reduce redundancy, which is especially relevant due to the use of multiple proteases, assembled contigs were filtered to eliminate duplicates and overlapping sequences. Starting from these initial contigs, an iterative scaffolding step was applied (up to 10 rounds), which progressively merges and extends sequences until convergence is reached. We consider it a critical methodological choice to set the minimum overlap to at least three amino acids to prevent erroneous contig generation.

#### Peptide Assembly With DBGs

In contrast to greedy assembly, the DBG method fragments each peptide sequence into overlapping subsequences, or k-mers. Each k-mer represents a node in the graph, and edges are defined by overlaps of k − 1 amino acids between adjacent k-mers. We implemented a weighted DBG strategy where edge weights correspond to k-mer frequency, addressing the specific challenges of *de novo* peptide assembly. The method employs a hierarchical assembly strategy. Initial linear paths, defined as contigs, are extracted by traversing unambiguous unitigs. Successively, we subject these contigs to an iterative Overlap-Layout-Consensus (OLC) refinement phase (51). In this step, contigs are treated as nodes in a higher-order overlap and are iteratively merged based on suffix-prefix overlaps exceeding a defined length, yielding the final set of consolidated scaffolds. Following the Overlap-Layout-Consensus refinement, the resulting scaffolds are ranked to prioritize sequences that are both structurally consistent and supported by high evidence. We compute a post assembly score for each scaffold using a linear combination of length and coverage metrics:

S=alpha∗ln(L)+beta∗w_mean+0.2∗w_min

where L is the amino acid length of the scaffold, w_mean denotes the mean k-mer coverage along the assembly path, and w_min corresponds to the minimum edge weight observed within the sequence. We applied a natural logarithmic transformation to the length term to ensure that the marginal contribution of sequence length decreases as the assembly grows, balancing sequence contiguity with structural confidence. This ensures that the ranking favors robustly supported scaffolds over excessively long, potentially chimeric artifacts. The mean coverage term acts as a direct measure of peptide abundance, prioritizing scaffolds that are supported by frequent spectral observations. Finally, to safeguard structural robustness, we incorporated the minimum edge weight with a penalty factor of 0.2. This component ensures that high scores are assigned only to sequences that maintain continuous experimental support without relying on tenuous, low-frequency connections. The final assembly output is presented as a ranked list, enabling downstream analysis to focus primarily on the highest-confidence protein candidates.

The parameters optimized for the DBG-based method included (1) the k-mer size used for graph construction, (2) the minimum overlap required to merge contigs into scaffolds, (3) the minimum weight threshold for edge filtering, and (4) the minimum sequence length threshold to retain valid contigs. Although k-mer size is a key parameter in DBG-based approaches, we established seven as the default value following a grid search, since for all sample types, similar performance was observed for k-mer sizes of 6 or 7, with dramatically reduced performance with any other size.

#### Clustering, Alignment, and Consensus Sequence Generation

We clustered assembled scaffolds using MMseqs2 (52) with a minimum identity threshold set to 0.85 and a coverage mode of 1 to merge near-identical sequences and reduce redundancy arising from overlapping or partially assembled contigs. A minimum identity threshold of 0.85 ensures that only sequences sharing at least 85% amino acid identity over the aligned region are grouped into the same cluster, thus preserving sequence specificity. With coverage mode 1, MMseqs2 computes the alignment coverage as the fraction of the target sequence that is aligned to the query:

Coverage=Numberofalignedresidues/Lengthoftargetsequence

For each resulting cluster, we performed multiple sequence alignment using Clustal Omega version 1.2.4 (53), with default settings. From each aligned cluster, we computed a position-specific scoring matrix (PSSM) by calculating the relative frequency of each amino acid at every aligned position, excluding gaps. This matrix is subsequently used to derive a representative consensus sequence. At each aligned position, we selected the amino acid with the highest frequency among the aligned sequences.

#### Parameter Optimization and Assembly Evaluation

To optimize the performance of the assembly algorithms, a systematic hyperparameter search was carried out using both grid search and DBG strategies. A structured set of parameters, including k-mer size, minimum overlap, size threshold, minimum edge weight, FDR, and refinement rounds, was explored by generating all possible combinations across predefined ranges. This exhaustive exploration produced 459 distinct configurations for each sample. To efficiently manage the computational workload, the search was executed in parallel using Python’s ProcessPoolExecutor, leveraging up to 64 workers (threads) concurrently. Rather than relying on coverage alone, we identified the optimal configuration using a composite score that integrates coverage, N50, total sequence count and mean identity.

#### Composite Score

To comprehensively assess assembly quality, we utilized a composite score integrating four complementary metrics: coverage, N50, scaffold count, and mean identity. We normalized each metric using Min-Max scaling, which linearly scales the original values to a range between 0 and 1. This normalization step ensures that all metrics contribute comparably to the final score, regardless of their original scale or units. The composite score for each assembly was calculated as the dot product between the vector of normalized metrics and the corresponding vector of weights, following the formula:

Compositescore=Σ_i(w_i×m_i)

where w_i denotes the weight assigned to metric i and m_i is the corresponding normalized value. For scaffold count, where lower values indicate improved contiguity, we utilized an inverted normalization (1 - normalized value) to ensure that assemblies with fewer fragments received higher scores.

We assigned weights based on the relative importance of each metric in reflecting key aspects of assembly quality. Coverage received the highest weight (0.35), as it represents the most critical indicator of the extent to which the input data support the assembled sequences. N50 was assigned a weight of 0.25, capturing contiguity. Scaffold count was weighted at 0.25 to penalize fragmentation. The mean identity weighted at 0.15, to reflect quality over quantity. Finally, we ranked assemblies by a descending composite score.

#### Benchmarking of InstaNexus with ALPS

To benchmark our assembly workflow with existing methods, we assembled proteins from our samples using the same peptide identifications with InstaNexus and ALPS (4) (2016/v1.0). We evaluated the assemblies using the metrics derived from mapping scaffolds to their ground-truth protein sequences, applying specific quality thresholds. N50 was defined as the shortest length needed to cover 50% of the total assembled length. Precision was calculated as the percentage of total scaffolds that mapped to the reference with a minimum identity of 40%, a threshold selected to exclude spurious noise alignments while retaining homologous segments. Mean identity measured the average sequence accuracy, computed exclusively on scaffolds mapping with an identity threshold ≥70% to focus the metric on reliably identified regions. High-confidence coverage represented the percentage of the reference protein sequence covered by scaffolds exhibiting ≥90% identity, ensuring that the metric reflects only highly accurate sequence reconstruction. We used these metrics to compute a combined assembly quality score (AQS) to reward high accuracy and penalize fragmentation:

AQS=100×(C_norm×I_norm×P_norm)/log10(N+9)

where C_norm, I_norm, and P_norm denote the normalized (0–1) values of coverage, mean identity, and precision, respectively, and N is the total number of scaffolds. The logarithmic term includes a regularization constant (+9) such that an ideal single-scaffold assembly (N = 1) yields a fragmentation penalty of 1, thereby normalizing the score range.

#### Pipeline Reusability and Repository Structure

We implemented InstaNexus as a fully modular Python package (https://pypi.org/project/instanexus/), allowing for direct installation via pip. To ensure cross-platform reproducibility, we provide dedicated Conda environments (environment.linux.yml and environment.osx-arm64.yaml) optimized for both Linux and macOS systems. The repository (https://github.com/Multiomics-Analytics-Group/InstaNexus) is organized to facilitate both execution and “quick start” documentation. The “src” directory contains the core logic of the InstaNexus package, while the directory docs/source/tutorials provide tutorials for the workflows. Furthermore, the “scripts” directory contains executable modules for critical tasks, including hyperparameter optimization, benchmarking, and secondary analysis. All dependencies and usage instructions are documented in the repository’s README files.

## Results

### InstaNexus, an Optimized Workflow for Direct Protein Sequencing

**Fig. 1:**
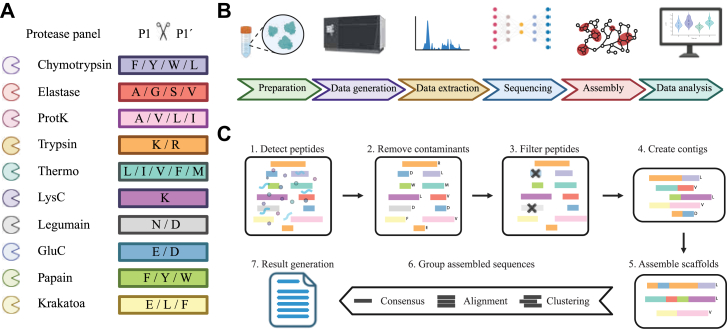
**Overview of the optimized direct protein sequencing workflow.***A*, a multiprotease panel covering a broad cleavage specificity range was established for enzymatic digestion. Proteases were selected and optimized to generate overlapping peptides, maximizing sequence coverage and reducing sample requirements. *B*, overview of the experimental and computational workflow: sample preparation data generation via mass spectrometry, peptide extraction, de novo sequencing using InstaNovo, assembly, and final data analysis. *C*, assembly computational pipeline: (1) removing contaminants, (2) processed de novo peptide predictions, (3) filtering of low confidence peptides, (4) assembly into contigs using greedy or DBG approaches, (5) scaffold formation, (6) alignment, clustering, and consensus generation, resulting in (7) a final set of candidate protein sequences. DBG, de Bruijn graph.

Our direct protein sequencing workflow InstaNexus starts with a multiprotease panel for enzymatic digestion prior to MS analysis (Fig. 1*A*). We included proteases covering a broad specificity profile to ensure the generation of diverse and overlapping peptides and to maximize compatibility with a broad sample repertoire. We tested 14 different proteases and optimized reaction conditions and incubation times individually for each protease. Thereafter, the performance was evaluated by the number of unique peptides detected through database searches using BSA as a model sample. Our optimized sample preparation workflow includes 10 proteases, each digesting the sample in separate reactions. The sample preparation is completed in less than 4 h, making the process highly time-efficient and cost-efficient. Moreover, only 2 to 10 μg of material is required per protease digestion, thus minimizing the sample amount needed for analysis. For peptide prediction, we selected InstaNovo as the foundation for *de novo* sequencing due to its high performance across a wide range of biological contexts (41). InstaNovo delivers accurate and generalizable peptide identifications, including non-tryptic peptides and modified residues, which are essential for robust downstream assembly (Fig. 1*B*).

InstaNexus assembles protein sequences using two complementary computational methodologies: a greedy assembler and a DBG-based assembler. These approaches independently generate overlapping peptide contigs, which can then be merged to produce longer sequence scaffolds, the term we use to describe meta-contigs or assembled contigs following the terminology in genomics. In the early stages of assembly, duplicates or fully contained contigs within longer ones are removed to reduce complexity. The remaining nonredundant contigs are then merged into scaffolds to further extend sequence length. The resulting scaffold space represents the candidate protein sequences. However, several scaffolds may still be partially overlapping or contain positional disagreements. To consolidate this space, we align scaffolds using ClustalOmega and perform clustering with MMseqs2. From each cluster, we generate a frequency matrix and derive a consensus sequence, reducing the scaffold pool to a minimal, high-confidence set of candidate sequences that cover the target sequence with high information content (Fig. 1*C*).

### InstaNexus Generates High-Quality Predictions and Sequence Coverage at Peptide Level

**Fig. 2:**
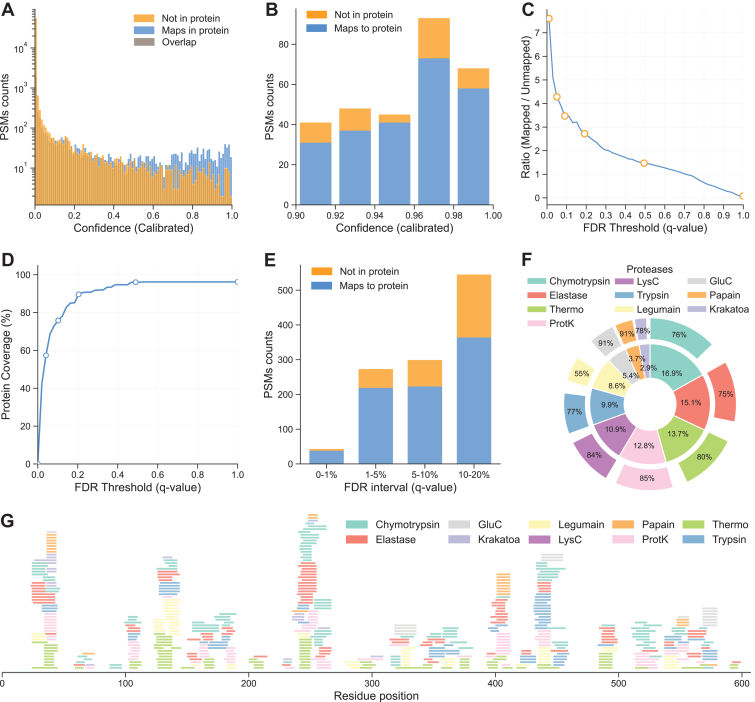
**Evaluation of peptide prediction quality and sequence coverage.***A*, distribution of peptide-spectrum matches (PSMs) across calibrated confidence scores, showing mapped peptides (*blue*), unmapped peptides (*orange*), and overlap (*gray*). *B*, percentage of mapped and unmapped peptides at high confidence intervals, highlighting the enrichment of mapped peptides at higher scores. *C*, ratio of mapped to unmapped peptides plotted against FDR thresholds (q-values). *D*, protein sequence coverage as a function of FDR threshold. Coverage increases rapidly at low q-values and reaches saturation, demonstrating that a permissive threshold (10 or 20%) still retains substantial sequence information. *E*, mapped and unmapped PSMs across discrete FDR intervals. The 0 to 1% interval contains predominantly correct identifications, whereas the 10 to 20% interval shows a marked increase in unmapped peptides (false positives). *F*, contribution of each protease to the total PSMs and mapping efficiency to the reference sequence. The inner segments represent the percentage of total PSMs attributed to each protease, while the outer segments reflect the fraction of those PSMs that successfully map to the reference protein sequence. *G*, mapping of peptides along the bovine serum albumin reference sequence, with each protease contributing complementary coverage. FDR, false discovery rate.

To benchmark and optimize the computational components of our workflow, we first applied InstaNexus to BSA, a standard protein with a well-characterized reference sequence. InstaNovo generates a large number of peptides with assigned confidence scores, capturing the probability of a correct peptide identification. The output peptides spanned a wide range of confidence levels, and further filtering was required to ensure data quality and accurate downstream analysis (Supplementary Fig. S1). The correlation between confidence scores and peptide identification accuracy highlighted InstaNovo’s ability to prioritize high-quality peptide candidates (Fig. 2, *A* and *B*). To validate these identifications, we applied Winnow (50) to control FDR in *de novo* peptide sequencing. To define an optimal filtering strategy, we quantified the ratio of mapped versus unmapped peptides across increasing FDR thresholds (Fig. 2*C*). We observed that the mapped/unmapped ratio decreased as the FDR threshold increased, reaching a turning point where noise began to outweigh signal. We next assessed the impact of FDR filtering on protein sequence coverage (Fig. 2*D*). Coverage increased rapidly at low q-values, exceeding 80% at 10% FDR and reaching levels greater than 90% around 20% FDR. Although the curve fully saturated at approximately 50% FDR, the marginal gain in coverage decreased significantly after the 20% threshold. By selecting a permissive FDR threshold of 10%, we retained substantial sequence coverage while minimizing the inclusion of low-confidence predictions. On BSA, this trend was further corroborated by analyzing PSM counts within discrete FDR intervals (Fig. 2*E*). Peptides identified within the 0 to 1% FDR interval were predominantly correct, whereas the 10 to 20% FDR interval showed a significant increase in unmapped spectra, representing potential false positives or peptides mapping to other proteins.

To evaluate the contribution of individual proteases to peptide detection and mapping accuracy, we analyzed their performance in terms of both total PSM yield and mapping efficiency (Fig. 2*F*). Proteases such as chymotrypsin, elastase, thermolysin, and proteinase K contributed not only with a high proportion of total PSMs but also showed mapping rates exceeding 75%, indicating high efficiency in generating detectable peptides. Trypsin and LysC also showed robust performance with high mapping efficiencies. In contrast, legumain yielded a lower mapping percentage (55%), which may reflect either broader cleavage patterns or reduced compatibility with InstaNovo, potentially leading to unmapped or ambiguously identified peptides. Proteases like papain and GluC demonstrated high mapping precision (>90%) but contributed a smaller fraction to the total peptide yield. We optimized reaction conditions such as incubation time, digest amount, and temperature for each protease included in our sample preparation panel (Supplementary Fig. S2, *A* and *B*). Mapping of low FDR (1%) peptides across the BSA sequence showed broad, complementary coverage resulting from combined protease-specific digestions (Fig. 2*G*), underscoring the value of our multiprotease strategy. Finally, we quantified the relative contribution of individual proteases to sequence coverage (Supplementary Fig. S2*C*), with distinct performance across our protease panel (Supplementary Fig. S2*D*). The included proteases contributed a substantial number of unique peptides that contributed to sequence coverage and generation of overlapping peptides that could later be used for assembly (Supplementary Fig. S2*E*).

### Hyperparameter Tuning Improves Scaffold-Level Coverage by Identifying Optimal Settings

**Fig. 3:**
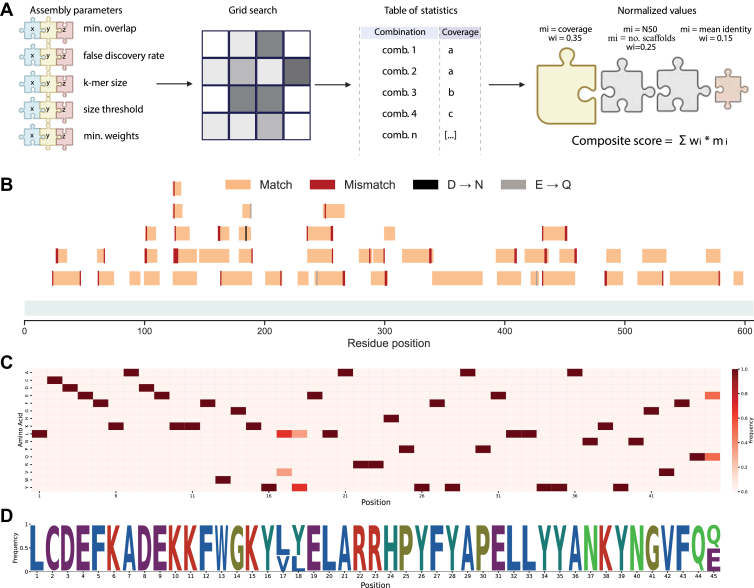
**Parameter optimization and consensus-based scaffold assembly.***A*, hyperparameter optimization for scaffold assembly. Assembly parameters were varied in a grid search, and combinations were scored using normalized metrics to identify optimal settings. *B*, mapping of assembled scaffolds to the bovine serum albumin reference sequence. Matches (*light orange*), mismatches (*red*), and known modification-driven substitutions (N→D, Q→E) are annotated. *C*, position-specific scoring matrix (PSSM) of amino acid frequencies across aligned scaffolds. *D*, sequence logo plot derived from the PSSM showing residue prevalence across the consensus.

After establishing high coverage and accuracy at the peptide level, we sought to optimize the parameters of our multistep assembly workflow to maximize candidate sequence quality (Fig. 3*A*). Using our BSA dataset, we systematically evaluated sequence coverage resulting from different parameter combinations using a grid search approach. After testing optimal values for six parameters, we visualized the impact of four key parameters: FDR, size threshold, k-mer size, and minimum overlap (Supplementary Fig. S3). Although the assembly process yielded multiple contigs, which correlated with the size of BSA, we achieved deep coverage and accurate sequence assembly across the full-length protein (Fig. 3*B*). Notably, the overall coverage would be even higher if the N-terminal signal peptide and propeptide, which is typically cleaved in the mature form of BSA, was not included in the reference sequence. Mapping analysis also revealed mismatches and frequent amino acid substitutions, in particular N→D and Q→E deamidation events compared to the reference sequence. We were able to construct a frequency matrix from each cluster containing aligned scaffolds, which demonstrated high confidence and discrimination across positions (Fig. 3*C*). From the position-specific scoring matrix, we derived a sequence logo plot to visualize the most likely amino acid candidates at each position within the generated sequence, which are in agreement with the reference sequence (Fig. 3*D*).

### InstaNexus Produces Long Contigs and Detects Hypervariable Regions in Nanobodies

**Fig. 4:**
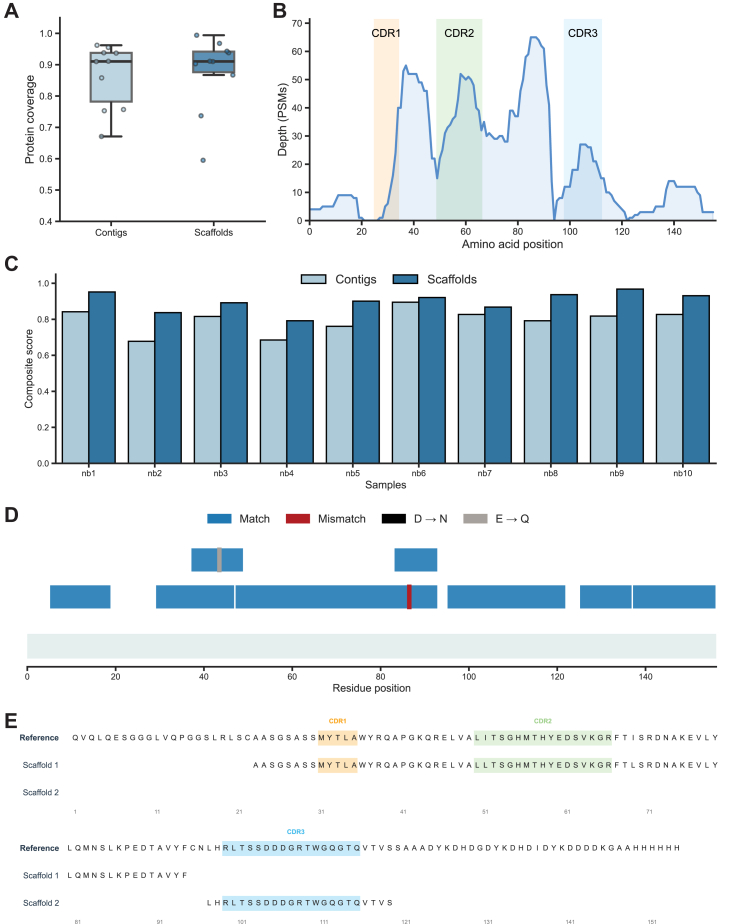
**Sequence reconstruction accuracy across nanobodies.***A*, coverage comparison between contig-based (*light blue*) and scaffold-based (*dark blue*) assemblies across the sequences for the nanobodies included in this study. *B*, PSM depth across the sequence for nanobody 6. The *y*-axis indicates the number of matching PSMs per amino acid position. All three complementarity-determining regions (CDR1-CDR3 shaded) are well covered, with CDR2 showing the highest signal intensity. *C*, composite scores per nanobody, comparing contig and scaffold-based assemblies. Scaffolds generally show improved scores across nanobodies. *D*, scaffold-to-reference sequence alignment for nanobody 6 showing matches (*blue*), mismatches (*red*), and sequence ambiguities (*gray bars*). *E*, consensus sequence comparison for nanobody 6, aligned to the reference. All three CDRs are accurately recovered. Notably, in scaffold 1, both CDR1 and CDR2 are recovered with maximal accuracy within a single scaffold. CDR, complementarity-determining region; PSM, peptide-spectrum match

Our method’s performance with BSA suggested potential for application to other proteins. Therefore, we set out to sequence a panel of 10 nanobodies. Overall, we achieved high maximum coverage across the majority of nanobodies (Fig. 4*A*), with comparable performance between contigs and scaffolds. We analyzed the PSM depth across the nanobody sequence, focusing on the CDRs (Fig. 4*B*). The three CDRs exhibited distinct coverage patterns, with CDR2 showing the highest and most consistent signal. In contrast, CDR1 was preceded by a marked drop in PSM depth, while CDR3 coverage remained moderate. To evaluate the quality of the assemblies, we developed a composite score that integrates coverage, N50, assembled sequence count, and mean sequence identity using a weighted normalization framework. Composite scores ranged from 0.68 (Nb3) to 0.97 (Nb10), with nanobodies 1, 9, and 10 exhibiting the highest values (Fig. 4*C* and Supplementary Table S2).

We selected nanobody 6 for detailed analysis as a representative case. Scaffold-to-reference mapping revealed a largely accurate alignment across the sequence range (Fig. 4*D*), with negligible or evidence of deamidation events. Similar results were observed across nanobody 6 at contig level (Supplementary Fig. S4, *A* and *B*); while the number of sequences was naturally higher compared to scaffolds, the assembly maintained excellent coverage and identity.

To gain deeper insight into region accuracy at the functional level, we directly aligned the scaffold for Nb10 against the reference sequence. Importantly, we were able to recover all three CDRs with complete accuracy in the first two scaffolds ranked by importance (Fig. 4*E*).

### An Accurate Workflow for Antibody Sequencing

**Fig. 5:**
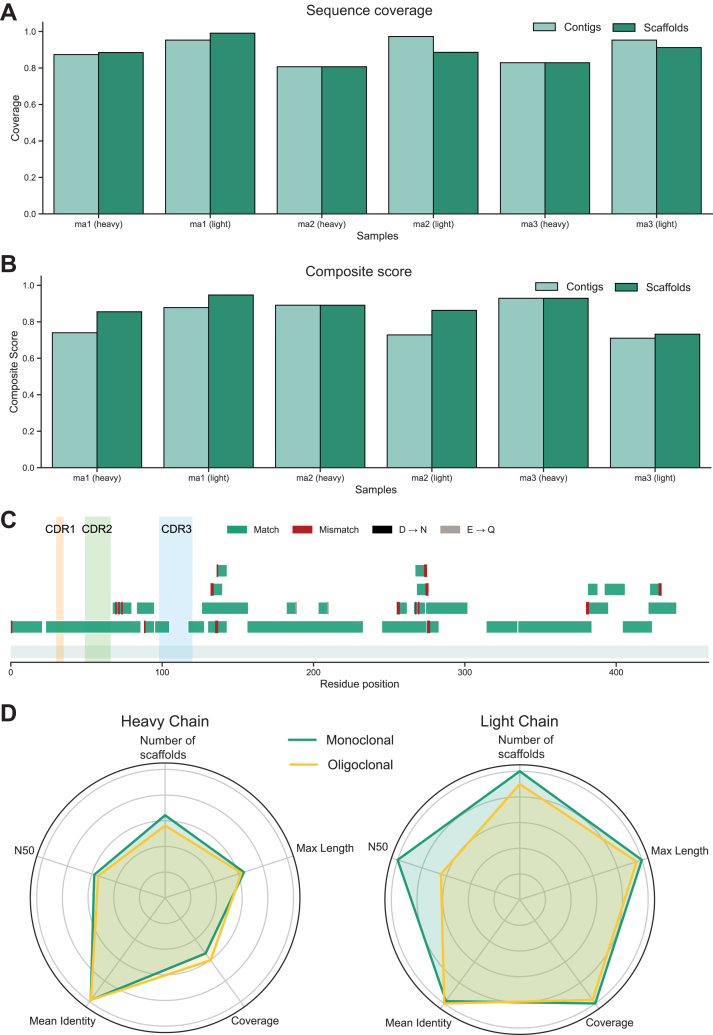
**Assembly performance across monoclonal and oligoclonal mAbs.***A*, maximum coverage for each monoclonal antibody chain (heavy and light), measured for contigs (*light green*) and scaffolds (*dark green*). *B*, composite scores for all monoclonal antibody chains, comparing contig and scaffold-level assemblies. *C*, scaffold-to-reference mapping for the heavy chain of monoclonal antibody 1 (mAb1), indicating sequence matches (*green*), mismatches (*red*), and deamidation substitutions. *D*, scaled performance metrics for mAb1 sequenced individually versus as part of the oligoclonal antibody mixture, shown separately for the light and heavy chains. Metrics include total sequences, maximum length, N50, coverage, and composite score.

We next assessed the performance of our workflow by sequencing three mAbs. High maximum coverage values were consistently observed across all mAbs, with comparable performance between contigs and scaffolds (Fig. 5*A*). Composite scores indicated robust sequence accuracy across mAbs, with mAb1 light chain and mAb3 heavy chain achieving the highest scores of 0.95 and 0.93, respectively (Fig. 5*B* and Supplementary Table S3). For detailed analysis, we selected the heavy chain of mAb1. Scaffold-to-reference mapping indicated accurate sequence reconstruction across the protein, enabling the complete recovery of CDR1 and CDR2, and partial recovery of CDR3 (Fig. 5*C*), characterized by frequent matches interspersed with sporadic mismatches. Typical deamidation events were also observed as previously. To further challenge our workflow, we pooled five distinct mAbs, including the three previously mentioned, and analyzed the resulting oligoclonal antibody mixture. To evaluate optimal parameters and maximum performance when the reference sequence was known, each mAb underwent a systematic optimization of assembly parameters. The composite score was then used to identify the optimal assembly conditions for each antibody (mAb1, mAb2, and mAb3). The average of these optimized parameters was subsequently applied to the dataset for the oligoclonal antibody mixture, enabling a direct comparison of performance metrics (Supplementary Table S3) between monoclonal and oligoclonal sequence generation. Compared to the oligoclonal mixture, mAb1 revealed distinct assembly profiles depending on the chain type (Fig. 5*D*). For the heavy chain, the overall assembly performance was similar between conditions, though closer inspection revealed subtle trade-offs. The monoclonal sample (green) demonstrated a slight advantage in the number of scaffolds and maximum length. Instead, the oligoclonal mixture (yellow) achieved marginally higher coverage. In contrast, the light chain showed more pronounced differences. We extended the comparison between monoclonal and oligoclonal to mAb2 and mAb3 (Supplementary Fig. S5, *A* and *B*). Overall, the monoclonals consistently improved structural assembly metrics (N50 and maximum length). In contrast, sequence coverage and mean identity remained largely comparable across conditions; notably, the oligoclonal mixture even achieved slightly higher coverage for the mAb3 heavy chain. We wondered whether digestion of a lower starting protein amount would produce similar results. To investigate this, we digested a total of 2 μg of mAb1 per protease digest with seven proteases, for a total of 14 μg of antibody. Using a more advanced MS setup, we found that this minimal amount still yields high sequence coverage and comparable chain assembly (Supplementary Fig. S6, *A* and *B*). Overall, monoclonal assemblies yield higher assembly metrics compared to the oligoclonal mixture, particularly for the heavy chains. This is not surprising, given the increasing sample complexity and ambiguity created when assembling proteins with mostly similar sequences.

### InstaNexus Can Sequence *de novo* Designed Binders

**Fig. 6:**
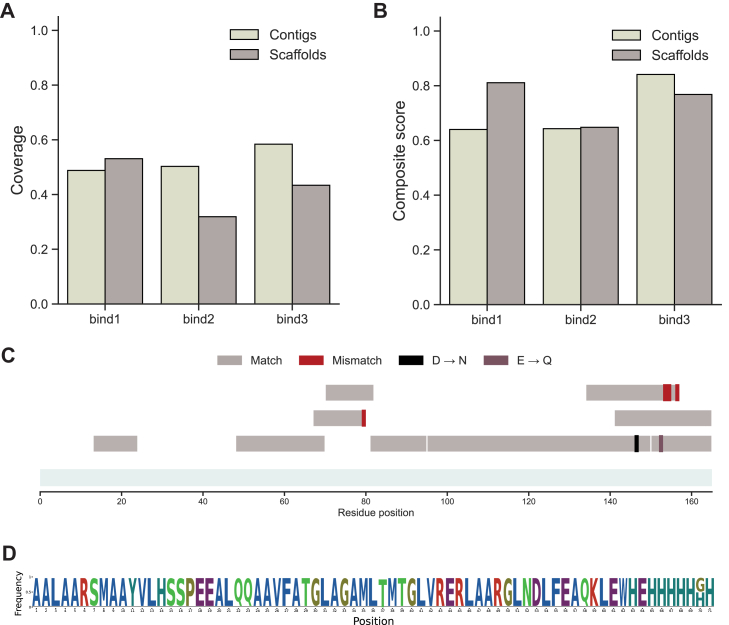
**Sequencing performance for de novo designed miBds.***A*, maximum coverage and composite scores for three designed miBds, comparing contigs (*light purple*) and scaffolds (*dark purple*). *B*, composite scores for three designed miBds, comparing contigs and scaffolds. MiBd3 shows the highest performance coverage and composite score. *C*, mapping of scaffolds to the miBd3 reference sequence. Matches (*dark purple*) and mismatches (*red*) are shown along the sequence. Mapping is equally distributed across the protein. *D*, sequence logo plot derived from a scaffold of miBd3 covering the final 71 residues. miBd, mini-binder.

Finally, we evaluated the capability of our workflow to reconstruct sequences of miBds. We analyzed three miBds designed *in silico* to target shared cancer antigens presented on major histocompatibility complex molecules. The overall maximum coverage observed for miBds was lower than for Nbs and mAbs. Among the three candidates, miBd3 emerged as the most robust sample. Although raw sequence coverage for contigs reached approximately 60% (Fig. 6*A*), miBd3 achieved a notably higher composite score (>0.80%) compared to miBd1 and miBd2 (Fig. 6*B* and Supplementary Table S4), showing higher quality assembly. Mapping of assembled scaffolds revealed a partial reconstruction (Fig. 6*C*). We observed a significant lack of coverage at the N terminus, with the first ∼15 residues completely missing, followed by another gap between residues 25 and 45. In contrast, the C-terminal region of miBd3 was recovered with high contiguity. However, we observed a higher rate of mismatches compared to other samples, especially in the C-terminal region, where deamidation events were also observed. We next examined the longest scaffold obtained for miBd3. Our consensus sequence visualized with a sequence logo plot derived from this scaffold confirmed recovery of a 71-residue stretch (Fig. 6*D*). Mapping scaffold coverage onto the structure revealed that some regions were not sequenced, likely due to sequence features rather than structural differences. In particular, the C-terminal region included a 6xHis-tag, which may be underrepresented due to difficulties in assembling low-complexity or repetitive sequences. Similarly, around position 40, the sequence includes two short, repeated stretches (AAARHVAAA), which might be filtered out under stringent thresholds.

### InstaNexus Produces Accurate Scaffolds and Minimizes Sequences for Experimental Validation

**Fig. 7:**
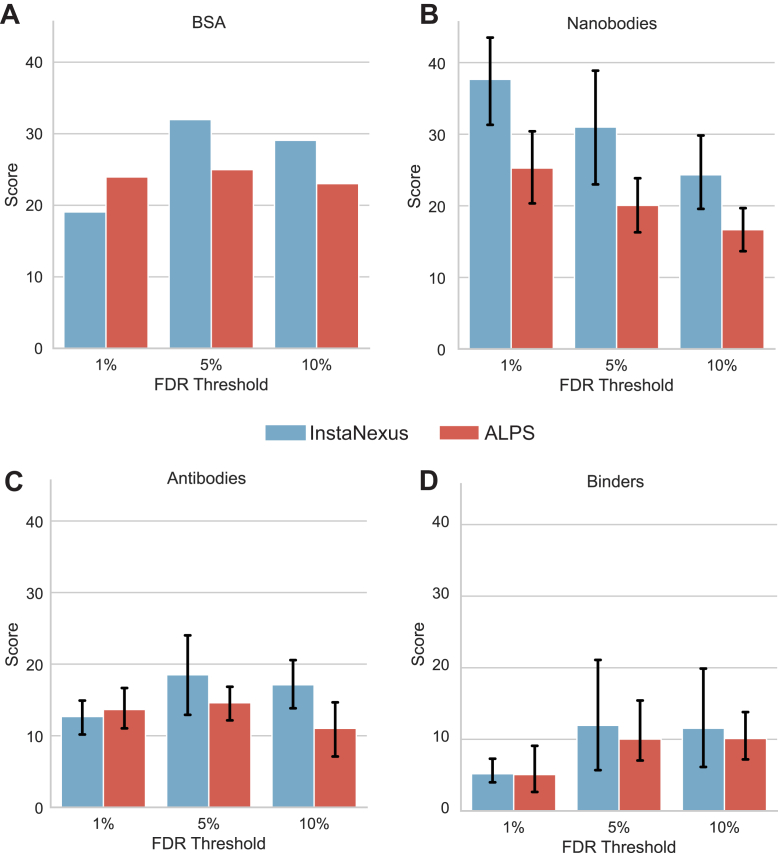
**AQS evaluation across different samples.** Comparison of assembly utility using the composite AQS metric for (*A*) BSA, (*B*) xnanobodies, (*C*) antibodies, and (*D*) binders at varying FDR thresholds (1%, 5%, and 10%). InstaNexus (*blue*) is compared against ALPS (*red*). Higher scores indicate a better balance of scaffold length, precision, and reduced fragmentation. Error bars represent the standard deviation. AQS, assembly quality score ; BSA, bovine serum albumin; FDR, false discovery rate.

An important consideration in direct protein sequencing is the number of sequences that need to be experimentally tested to confirm the putative protein assembly. This depends on the accuracy of scaffolds and the final number of scaffolds to be considered. Following the analysis of sequence coverage across FDR thresholds (Supplementary Fig. S7), we evaluated our assembly process by comparing our method with the ALPS assembler on our *de novo* peptide sequencing identifications. We aimed to quantify the balance between completeness, precision, and conciseness using the AQS (see Experimental procedures). InstaNexus consistently achieves superior or comparable AQS values across the samples tested (Fig. 7). Conversely, ALPS tends to produce a higher ratio of fragmented or inaccurate sequences (lower scores at comparable FDRs), which inflates potential experimental burden. Extended performance evaluations of each of the metrics used, including detailed comparisons of sequence coverage, accuracy, contiguity (N50), and number of scaffolds, support this observation (Supplementary Figs. S8 and S9). Overall, InstaNexus yields a more concise list of high-confidence candidates, significantly reducing the complexity of downstream screening.

## Discussion

Despite the critical need for direct protein sequencing in diverse applications in biotechnology, current approaches face significant challenges including complex laboratory protocols and data acquisition, and require large sample amounts as well as heavy manual sequence inspection. In addition, there is inherent difficulty in generating sufficiently long and contiguous peptide sequences, and the overall absence of integrated, end-to-end workflows capable of managing this assembly complexity effectively. Our work presents an optimized workflow for direct protein sequencing, combining an improved laboratory protocol and a new bioinformatic pipeline to assemble protein sequences with near complete coverage with only a few contigs.

Our sample preparation protocol leverages a diverse set of proteases to generate highly overlapping peptides, critically facilitating robust protein sequencing. The use of multiple proteases to increase peptide diversity and improve sequence coverage is well established in bottom-up proteomics. Previous studies have demonstrated that combining proteases with orthogonal cleavage specificities substantially improves sequence coverage and protein characterization compared to tryptic digestion alone, both at the proteome scale and for targeted protein analysis (37, 54, 55, 56). In this work, we systematically evaluate and optimize a protease panel specifically for *de novo* peptide sequencing and downstream assembly and integrate this optimized sample preparation strategy into a reference-free protein sequencing workflow. A recent study used the proteases Krakatoa and Vesuvius to generate overlapping peptides for antibody sequencing and demonstrated higher accuracy compared to using single protease digestion, validating the selection of these proteases in our panel (38). Crucially, our optimized sample preparation approach employs consistent buffer compositions wherever possible and limits digestion times to 4 h. This enables high-throughput preparation of samples, automation of nearly all steps before MS analysis, and result generation within a single day. Furthermore, since our *de novo* sequencing approach does not depend on protease specificity, protease digests from the same sample can potentially be pooled after quenching. Such a multiplexing strategy would reduce both instrument time and analytical costs.

To identify peptides in the InstaNexus workflow, we utilized a leading *de novo* peptide sequencing model and developed a novel assembly workflow that integrates two complementary assembly strategies (greedy and DBG) to extend contig length and improve coverage. We evaluated the identifications from the *de novo* peptide sequencing results and established a methodology where true hits reliably outnumbered false positives while retaining near-complete coverage of target protein sequences. Although both assembly methods share certain parameters, such as identity thresholds and minimum length filters, the greedy strategy offers greater flexibility in handling overlaps, particularly beneficial in noisy or low-coverage datasets. However, this flexibility increases its dependence on parameter tuning. Our assembly pipeline contained few complementary parameters to optimize that might be uniquely different based on the several protein classes that we tested. Our grid search optimization strategy allowed us to optimize the parameters for assembly methods, presenting an optimized scaffold workflow that can be applied to unknown sequences of the same modality. The scoring method we developed considers multiple samples and emphasizes additional metrics about sequence assembly rather than relying solely on coverage, integrating coverage with the quality and number of contigs instead. To support broader applicability, we defined a set of optimal parameters for contig and scaffold assembly. These parameters showed minimal variation across different sample types, allowing us to propose a generalizable, consensus configuration (summarized in Supplementary Tables S5 and S6). Researchers can use these default settings when applying the workflow to new or unknown samples, particularly in cases where reference-based optimization is not feasible.

These design features distinguish our workflow from existing tools, which offer valuable but more constrained strategies for peptide assembly. MuCS (57) and ALPS (4) both employ DBG-based approaches, but MuCS lacks parameter optimization and false-positive control, while ALPS depends on homology-based inputs, limiting purely *de novo* applicability. Stitch (30) performs template-guided assembly and is well suited for antibody profiling or other proteins for which homologous reference sequences are available, but it depends on user-provided templates and is therefore not designed for reference-free assembly. In contrast, our workflow is reference-free, parameter-optimized, and broadly generalizable across protein types and sample contexts.

A remaining limitation in this setting is FDR calibration for de novo PSMs. Although Winnow enables decoy-free error control and substantially improves over raw model confidence alone, purified single-protein experiments represent a partially out-of-distribution regime relative to its current training domain, which can lead to imperfect calibration. In practice, applying more stringent q-value thresholds increases precision but substantially reduces peptide yield and sequence coverage, limiting assembly coverage. For this reason, we intentionally apply a permissive peptide-level FDR threshold to maximize overlap density and assembly connectivity, while downstream DBG weighting and consensus-based assembly mitigate the influence of isolated false or partially correct PSMs.

In the MS analysis of this work, we only utilized HCD fragmentation, which could be supplemented with other fragmentation methods to produce complementary ion series and side chain fragment ions that could potentially increase assembly accuracy and consensus sequence confidence (37, 58, 59). Such fragment ions are primarily generated under other fragmentation schemes such as electron transfer dissociation, and can improve residue-level resolution and sequence coverage (60, 61). However, in this study we focus on HCD fragmentation, which is best supported by current InstaNovo releases and training data and is widely available across mass spectrometry platforms, enabling broad adoption of the workflow in standard proteomics laboratories. Another approach that could be considered would be to use offline fractionation of individual samples or digest pools to increase detection sensitivity. Both of these approaches, however, would increase sample preparation complexity and analytical costs. Another shortcoming of the methodology used in this work is that, due to their isobaricity, our workflow is unable to discern between isoleucine and leucine, which are therefore treated as interchangeable when mapping assembled contigs to reference scaffolds. While this can increase the number of candidate sequences that need to be validated experimentally, manual inspection of scans can provide additional information about the identity of the residue in a given position via the detection of side chain fragment ions (31).

In addition to the above limitations, it must be noted that although our workflow consistently produces long contigs for all tested samples, achieving a complete protein sequence with absolute confidence still remains elusive. However, the high coverage and contiguity of target sequences produced by our approach demonstrate the potential to provide truly actionable biological information. Although making a single complete and confident assembly of an antibody is currently lacking, comparative analysis of the generated consensus sequences with known reference sequences yielded in most samples 100% accuracy in the CDRs of mAbs and Nbs. Challenges also remain with complex samples, such as oligoclonal and polyclonal protein mixtures, where multiple proteins are present. Nevertheless, our method achieved promising results even in these cases, with performance comparable to that observed for individually assembled mAbs. This challenge was further amplified by the high sequence similarity among mAbs within the mixture, making accurate differentiation inherently more difficult. Other contributing factors to reduced performance with mAbs could be their multichain nature and the potential for extensive glycosylation in the heavy chain, a posttranslational modification that could be incorporated by future *de novo* peptide sequencing models to improve assembly performance. In addition, insufficient separation of the mAb chains during sample preparation might also contribute to reduced digestion efficiency, further impacting performance.

Despite the above-mentioned challenges, the successful application of our methodology to mAbs represents a significant step toward broader use cases, such as rapid antibody discovery and immune repertoire profiling. For *de novo* designed proteins, direct sequencing enables verification of design fidelity and confirmation of expression. By subjecting binder or antibody pools to selective pressure or target screening and applying *de novo* sequencing, researchers would potentially identify and characterize functional binders with high throughput and quantitative accuracy similar to display technologies (17), but without the need for prior tagging, library construction, or other molecular biology techniques. This opens the door to direct selection, screening, and sequencing of large antibody and binder libraries at the protein level.

Looking ahead, the next frontier in direct protein sequencing will likely involve its application to increasingly complex biological samples. Although this will likely require improving the performance of identification and addition of steps in preparation and data acquisition of our pipeline, the fundamental framework presented here remains broadly applicable and generalizable. Future work could focus on extending this methodology to complex samples and biological systems, enabling sequencing of polyclonal antibody mixtures. It would also unlock applications in metaproteomics, enabling sequencing of complex cellular communities, or sequencing novel enzymes for biotechnological applications. Improvements in *de novo* sequencing models that offer increased accuracy and widely expanded support for diverse posttranslational modifications may further enhance our ability to directly sequence proteins by improving alignment accuracy and coverage (40, 62). Together with more comprehensive sample preparation and MS technologies, complete and high-fidelity protein sequencing methodologies may emerge, which can possibly be used for the sequencing of complete proteomes without the need for reference databases.

## Data Availability

The InstaNovo checkpoint has been trained on the ProteomeTools datasets, Parts I-III and can be found in the ProteomeXchange Consortium via the PRIDE partner repository with identifiers PXD004732 (Part I), PXD010595 (Part II), and PXD021013 (Part III). The proteomics datasets and database search results generated in this study for BSA, Nbs, mAbs, and miBds have been deposited in PRIDE (63) under dataset identifier PXD066160. Reviewers can access the dataset with username: reviewer_pxd066160@ebi.ac.uk and password: TeperKWxjeqp. Supplementary data supporting the data preprocessing and analysis performed can be found on Zenodo with https://doi.org/10.5281/zenodo.16417501. The structure of miBd NY1-B04 was resolved and is publicly available in the Protein Data Bank (64) (PDB-ID: 9NNF).

## Code Availability

Inference code and model checkpoints for the base InstaNovo model are available at the InstaNovo GitHub repository https://github.com/instadeepai/instanovo. The InstaNexus pipeline and code can be found in the GitHub repository https://github.com/Multiomics-Analytics-Group/InstaNexus. All dependencies are documented in the repository, and the source code is released under the GNU General Public License v3.0 (GPLv3).

## Supplemental data

This article contains supplemental data.

## Conflict of Interest

J. D. and J. V. G. is an employee of InstaDeep, 5 Merchant Square, London, United Kingdom. The other authors declare no competing interests.
