# Supplementary figures and images for: Generalizable Direct Protein Sequencing With InstaNexus
Source: Mol Cell Proteomics. 2026 Mar 2;25(4):101547. doi: 10.1016/j.mcpro.2026.101547 (PMC13084398; doi:10.1016/j.mcpro.2026.101547)

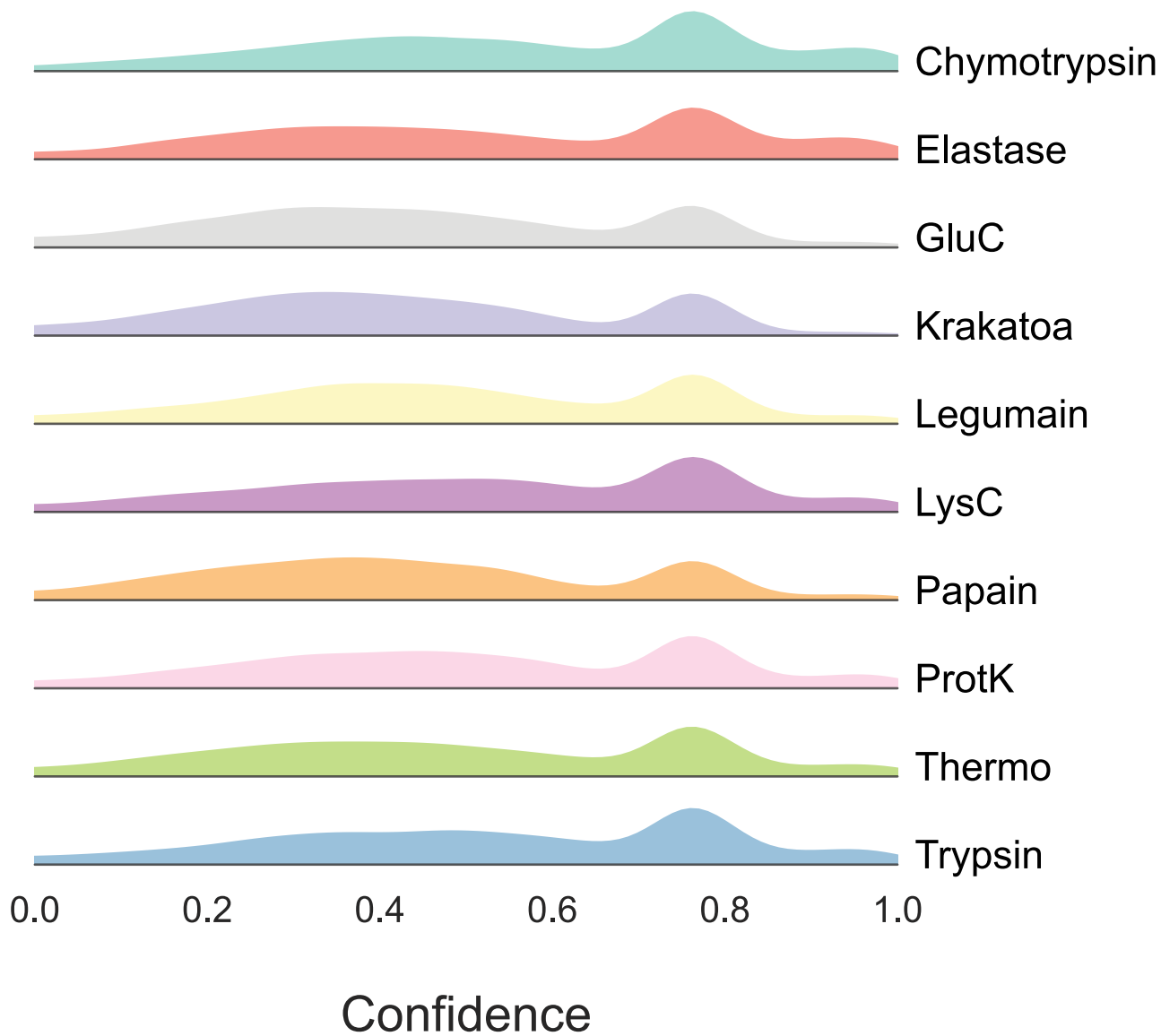

Supplement: Supplementary Figure 1 [file mmc1.pdf]

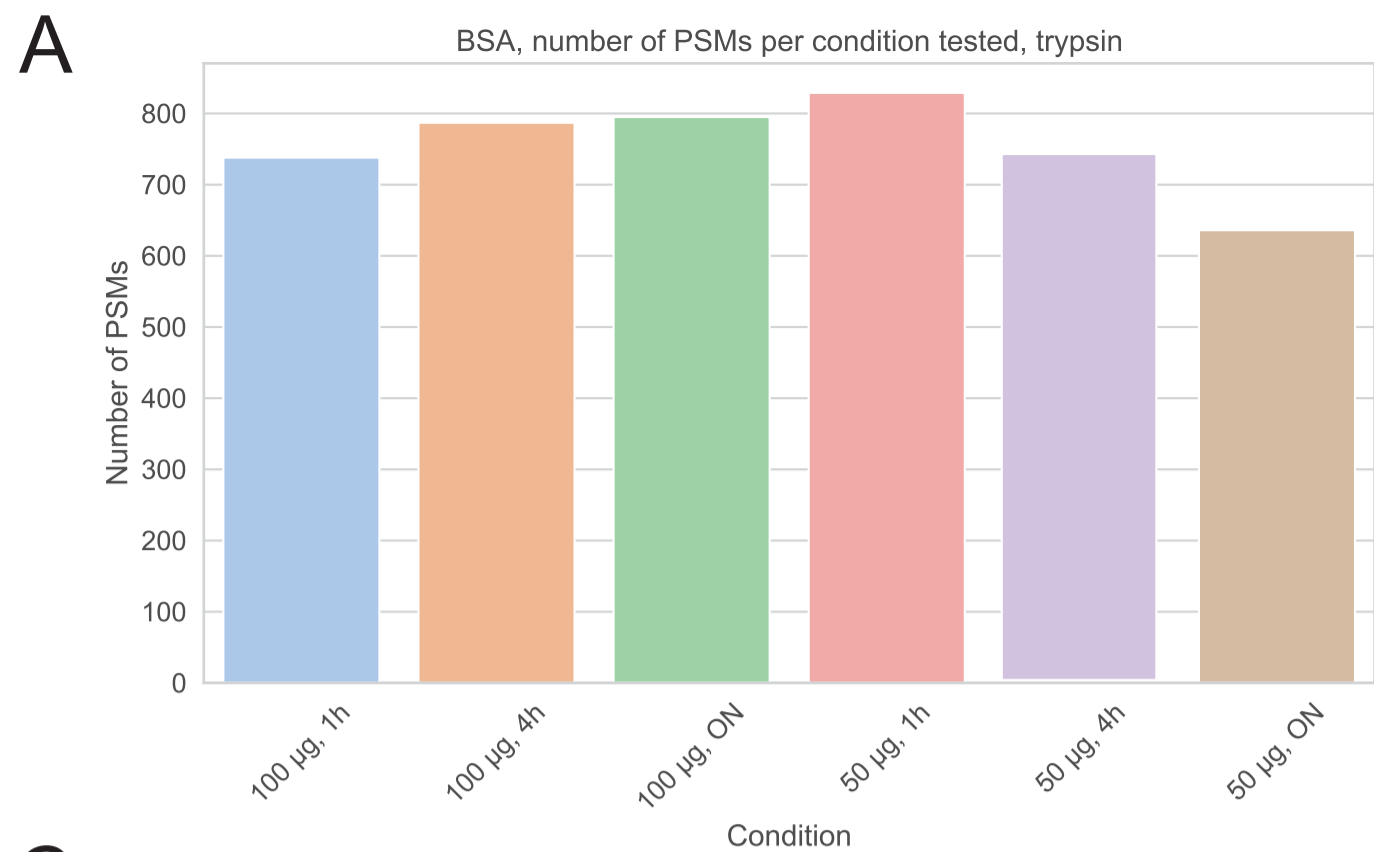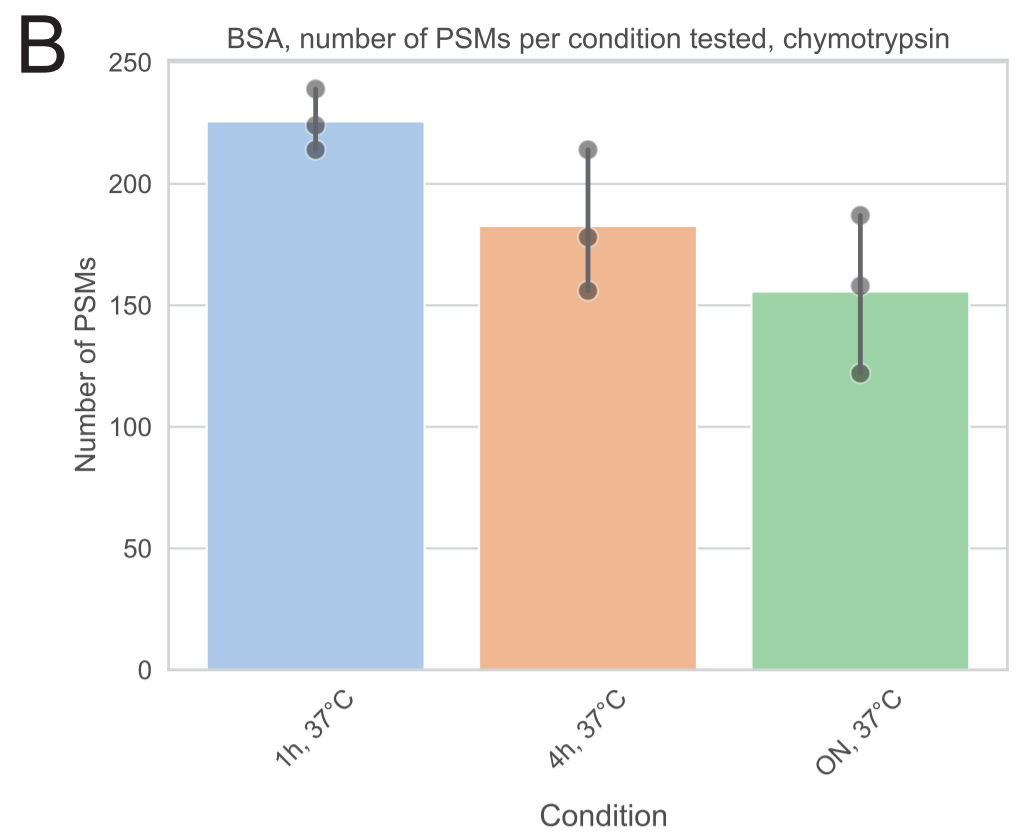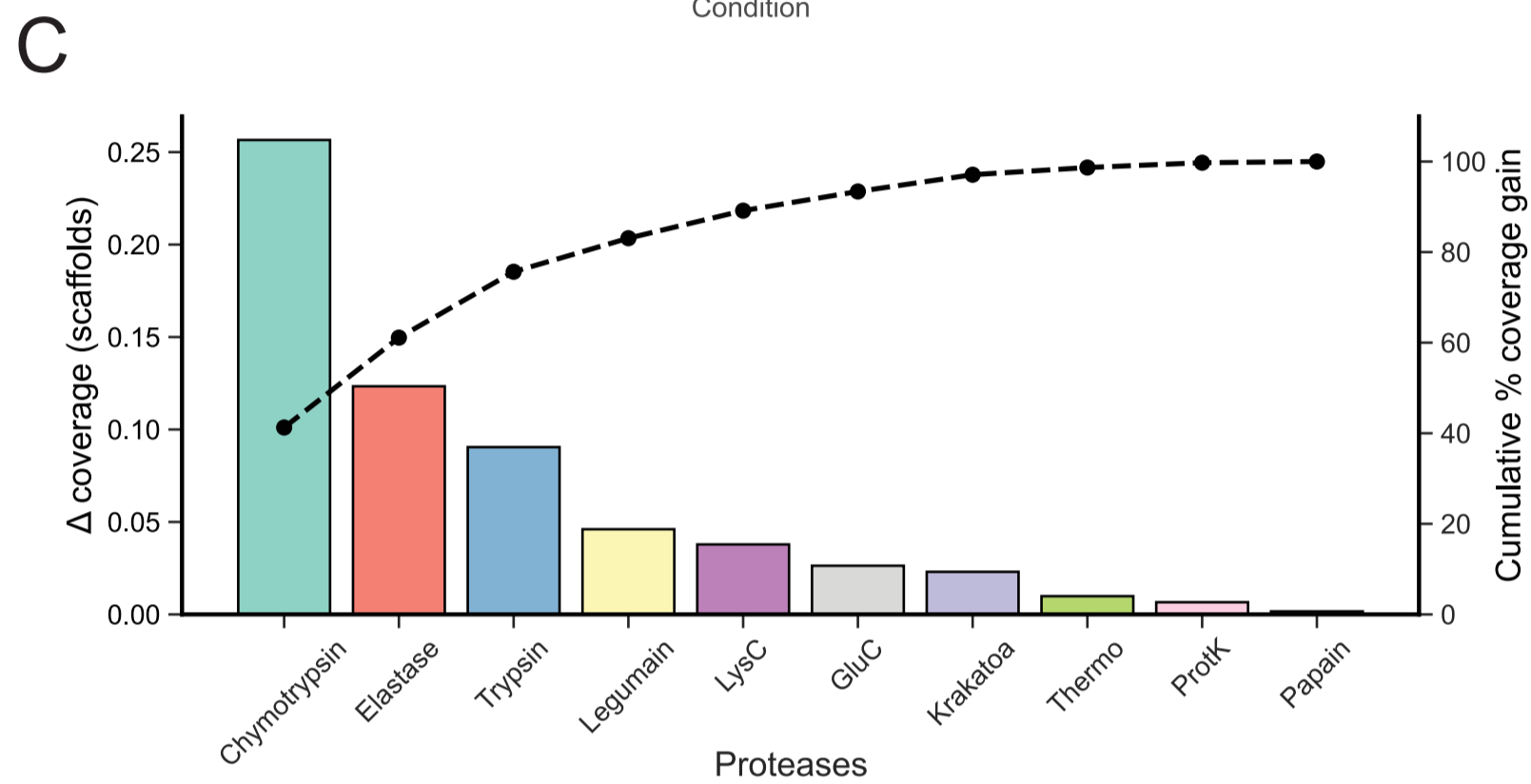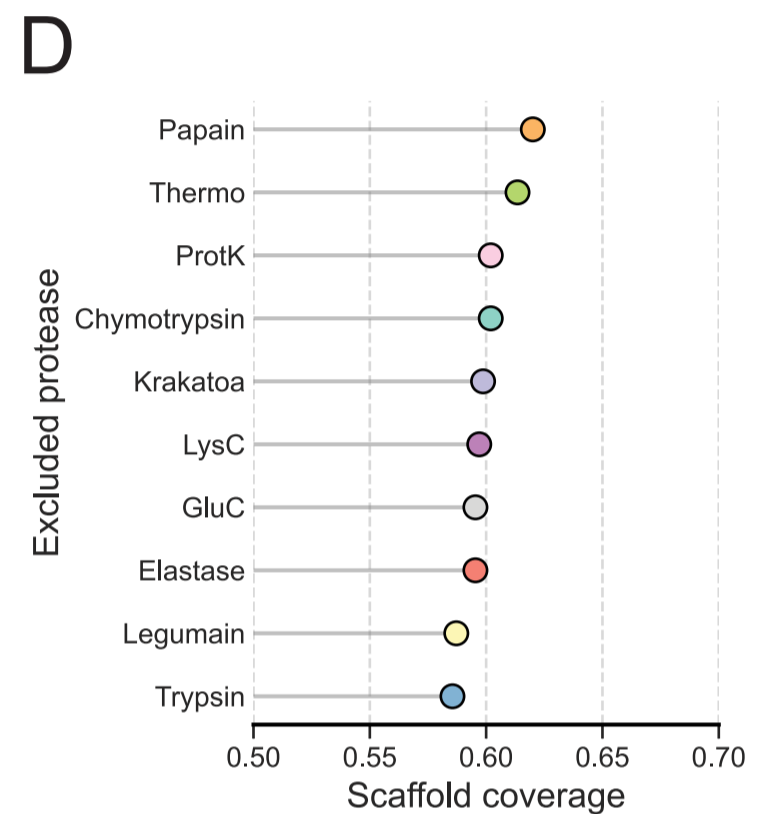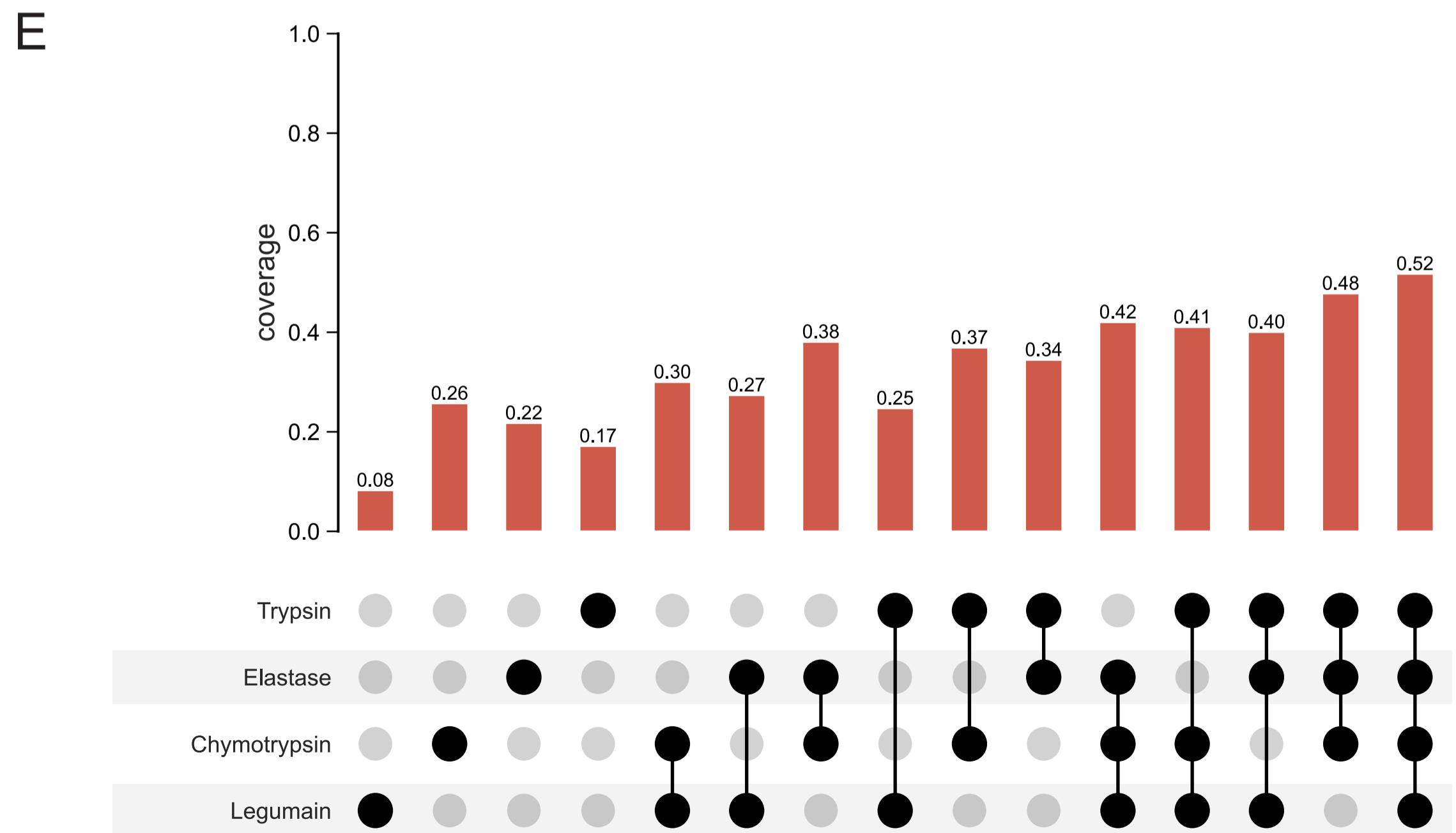

Supplement: Supplementary Figure 2 [file mmc2.pdf]

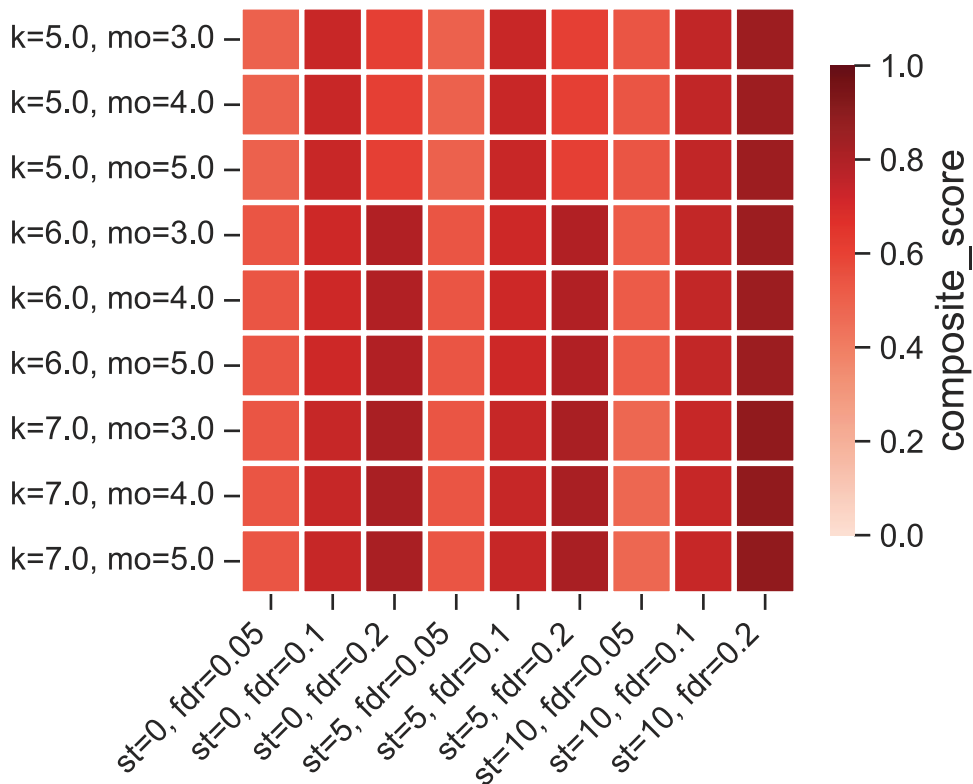

Supplement: Supplementary Figure 3 [file mmc3.pdf]

**A**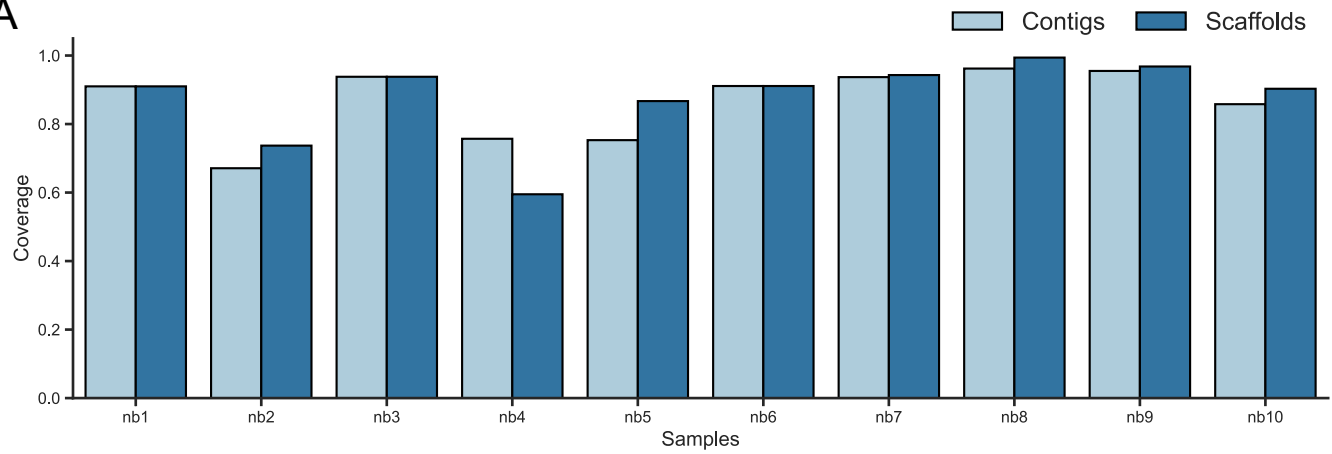**B**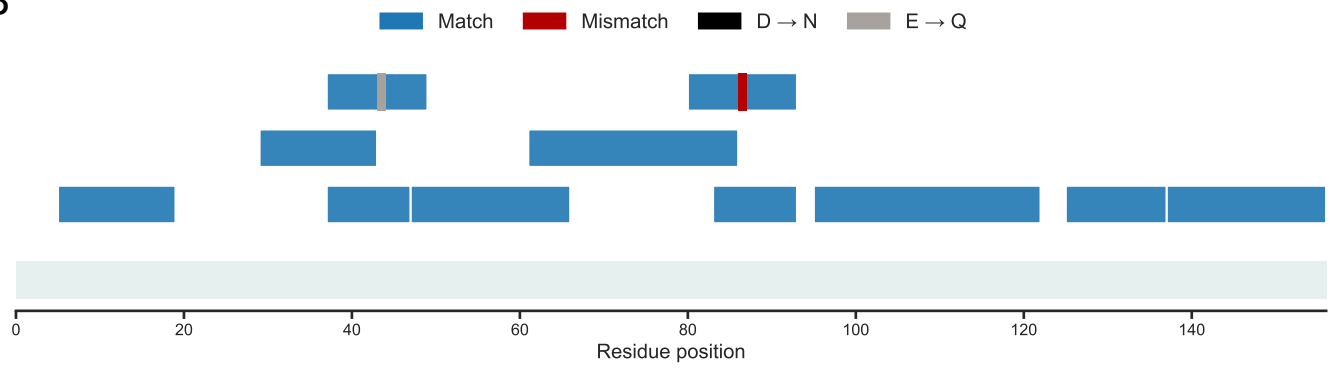

Supplement: Supplementary Figure 4 [file mmc4.pdf]

A

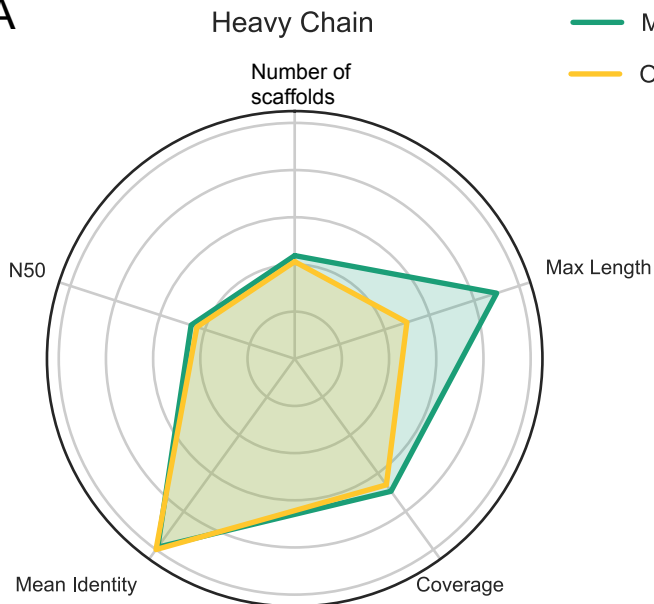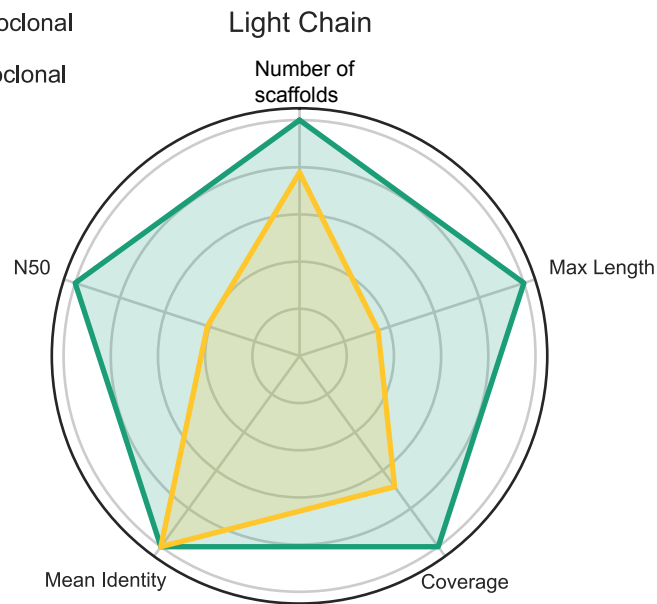

B

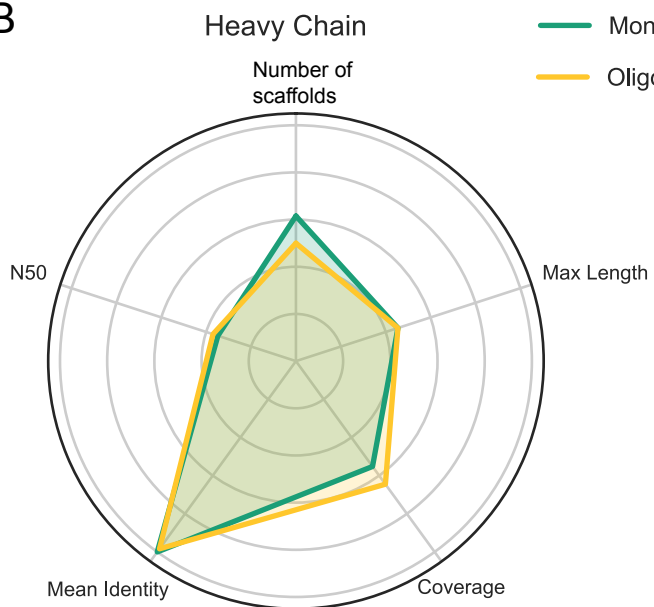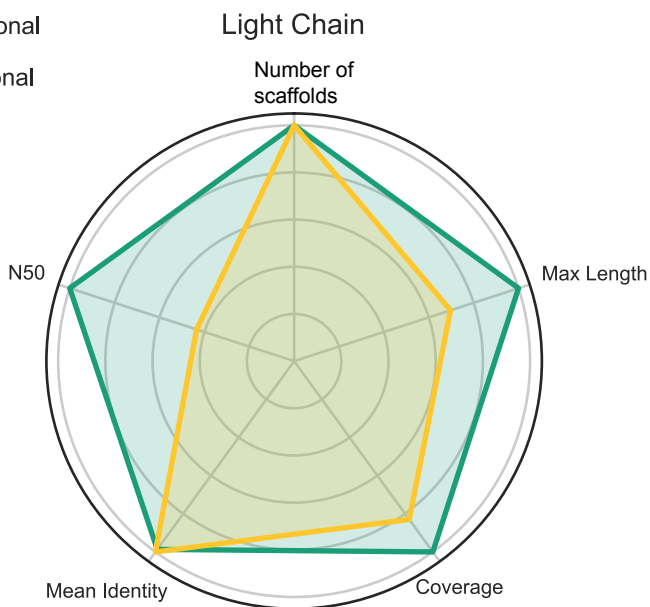

Supplement: Supplementary Figure 5 [file mmc5.pdf]

A

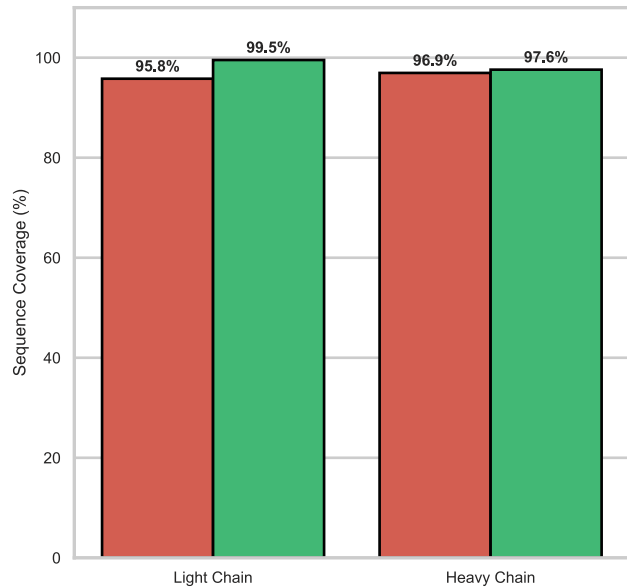

B

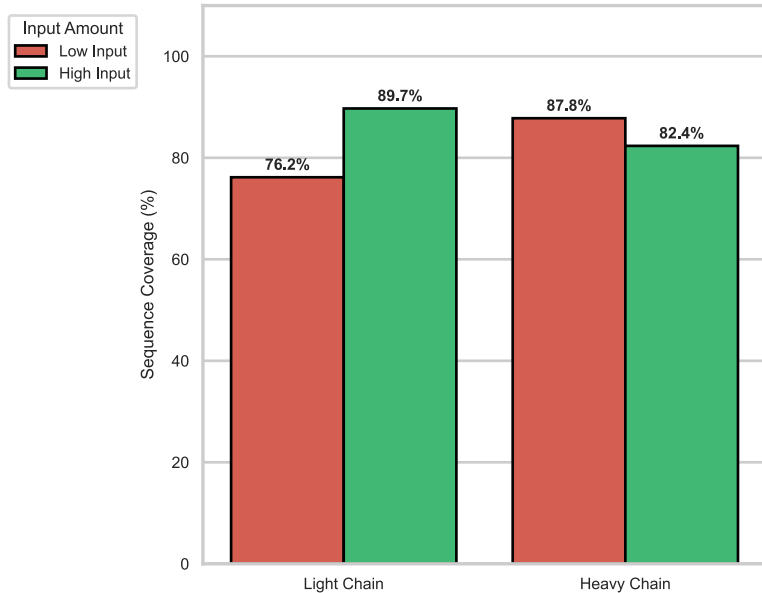

Supplement: Supplementary Figure 6 [file mmc6.pdf]

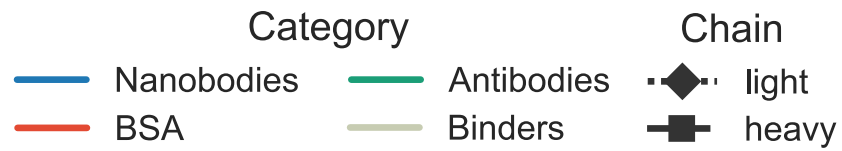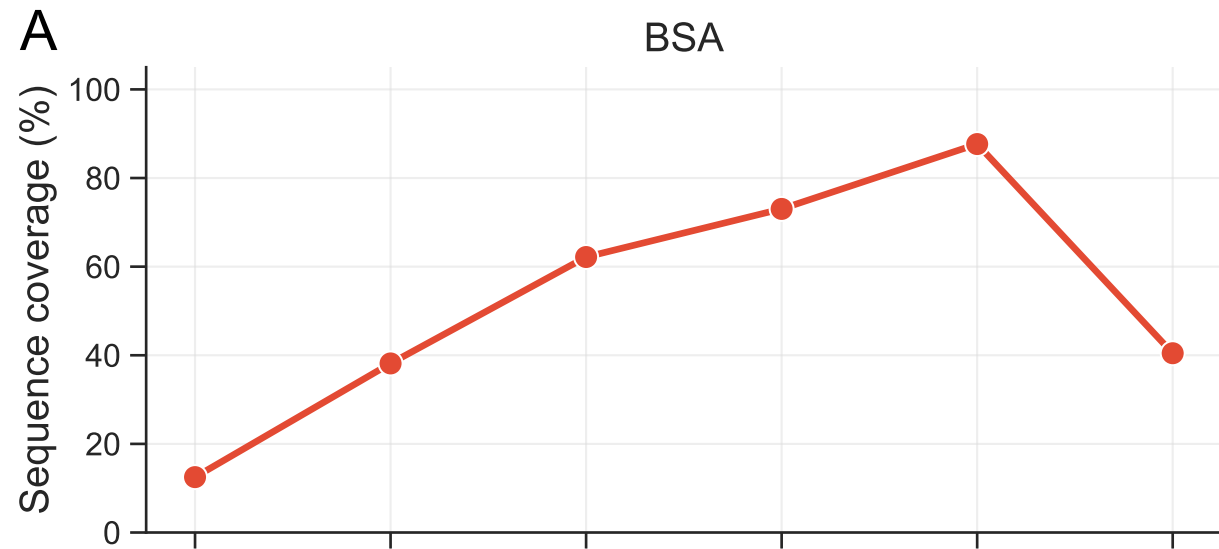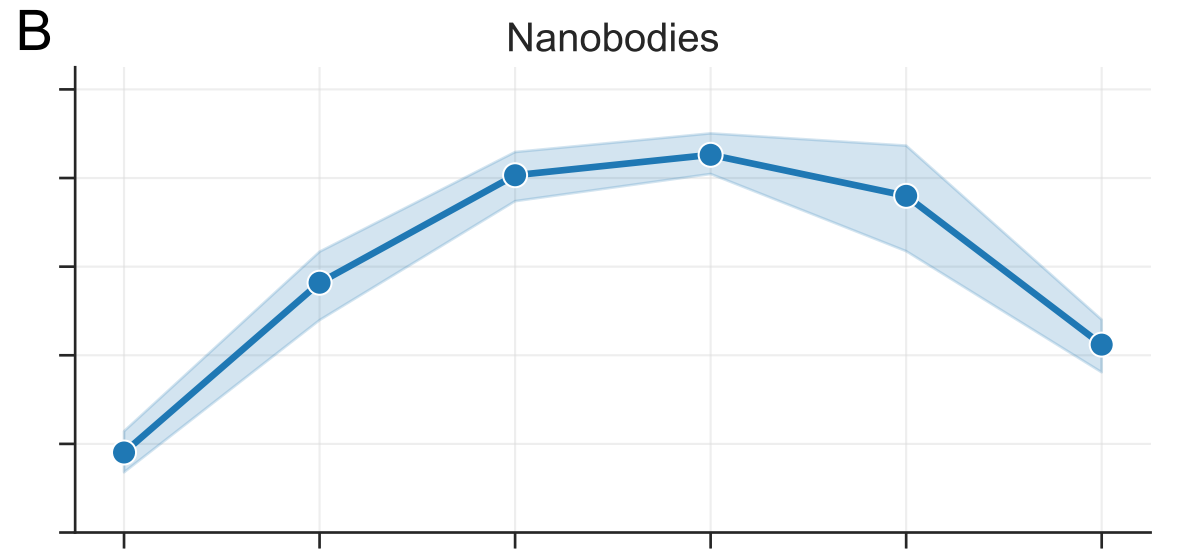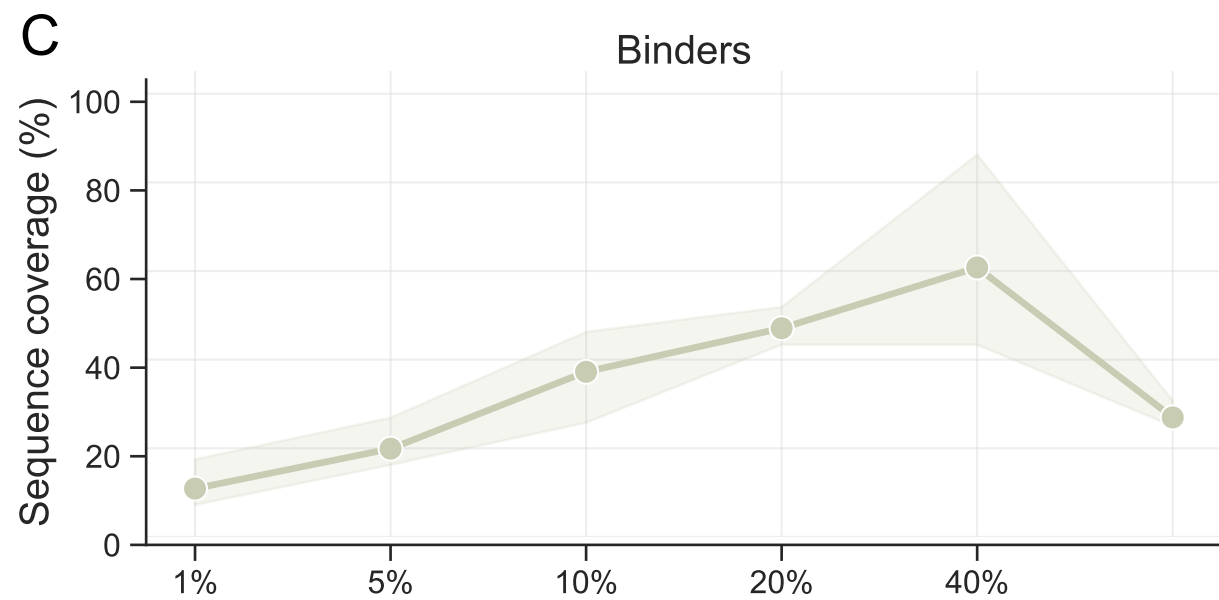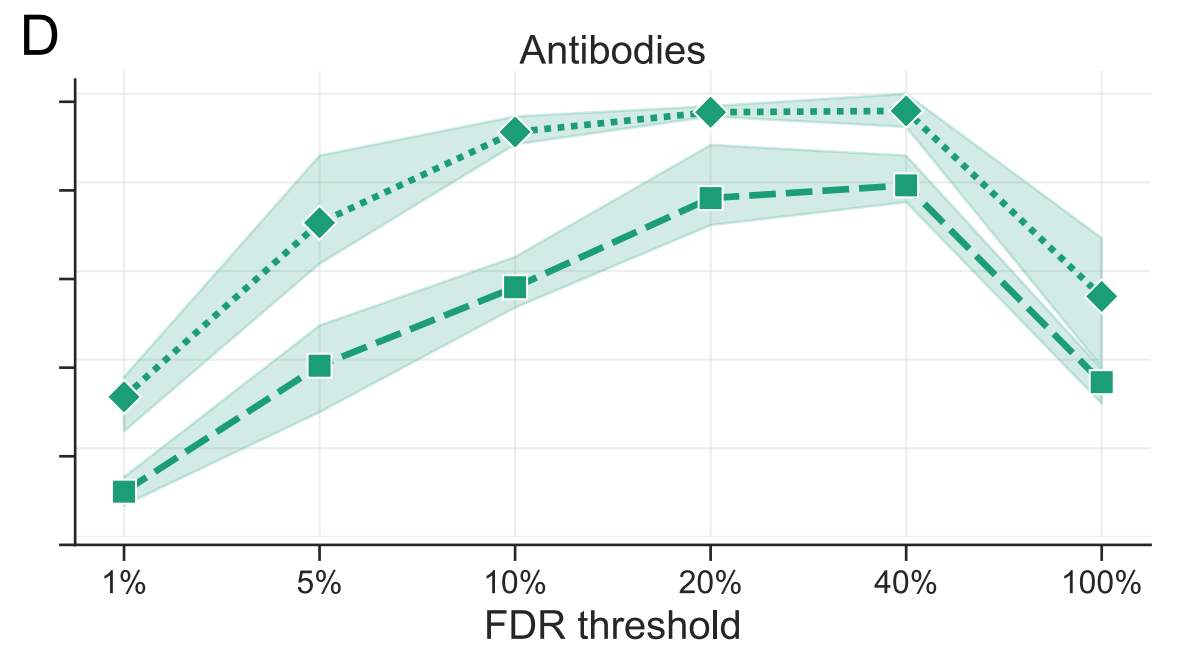

Supplement: Supplementary Figure 7 [file mmc7.pdf]

**A**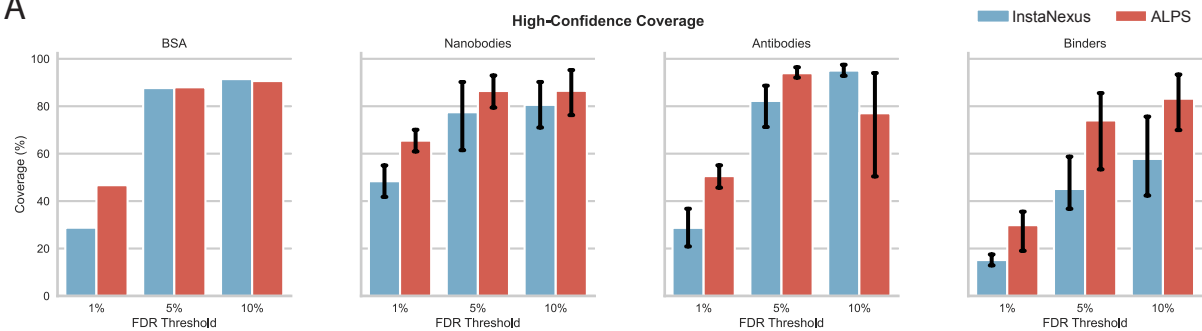**B**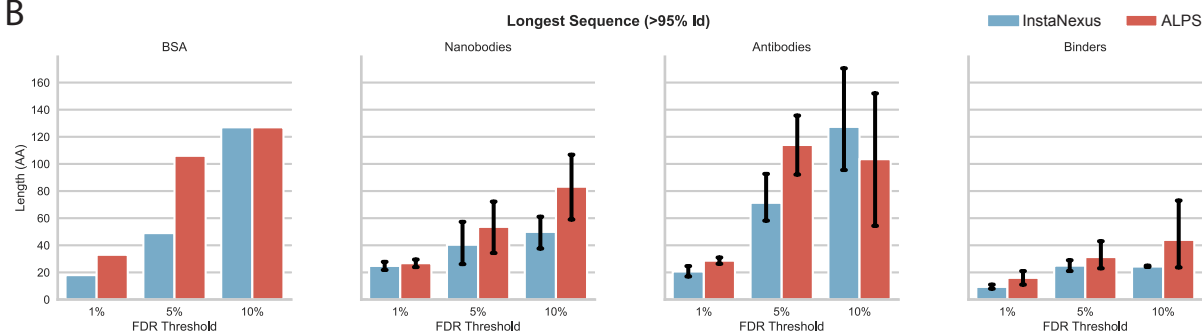**C**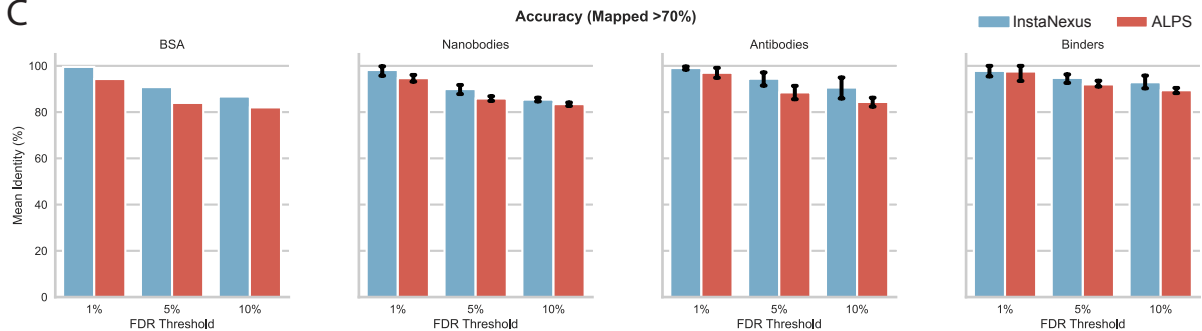

Supplement: Supplementary Figure 8 [file mmc8.pdf]

**A**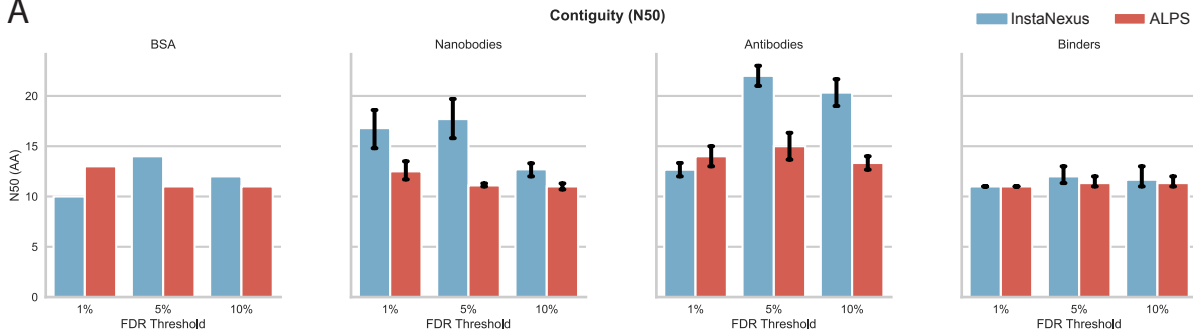**B**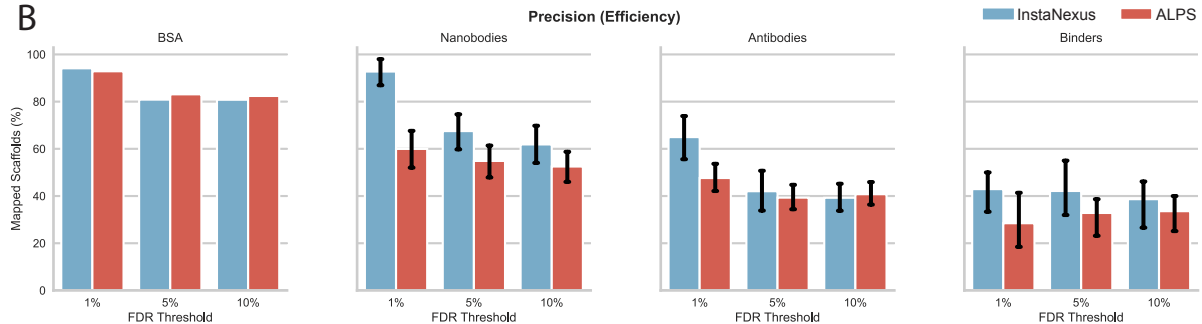**C**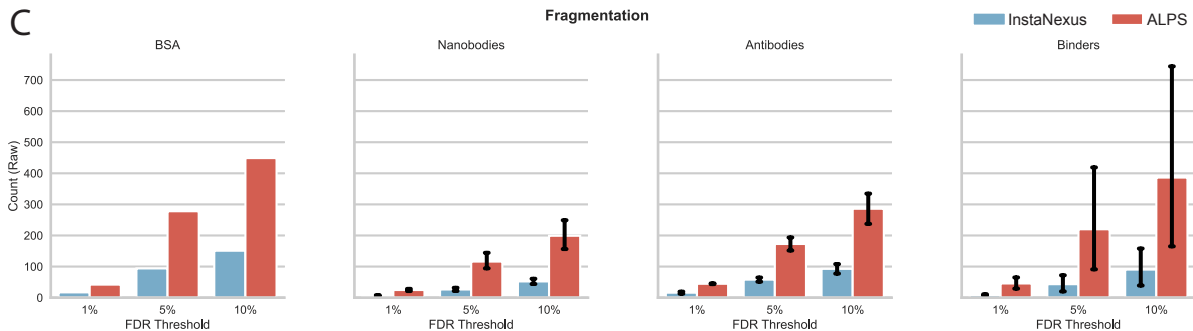

Supplement: Supplementary Figure 9 [file mmc9.pdf]
