# Supplementary material for: Generalizable Direct Protein Sequencing With InstaNexus
Source: Mol Cell Proteomics. 2026 Mar 2;25(4):101547. doi: 10.1016/j.mcpro.2026.101547 (PMC13084398; doi:10.1016/j.mcpro.2026.101547)
Supplement: Supplementary Data [file mmc10.docx]

# **Supplementary information**

# **Generalizable direct protein sequencing with InstaNexus**

# Marco Reverenna ^1^, Maike Wennekers Nielsen ^2^, Darian Stephan Wolff ^2,3^, Jemma Daniel ^4^, Elpida Lytra ^2,5^, Suthimon Thumtecho ^2^, Pasquale D. Colaianni ^1^, Anne Ljungars ^2^, Andreas H. Laustsen ^2^, Erwin M. Schoof ^2^, Jeroen Van Goey ^2^, Timothy P. Jenkins ^2,5^, Marie V. Lukassen ^2^, Alberto Santos ^1*^ and Konstantinos Kalogeropoulos ^2,5,6,7*^

^1^ Novo Nordisk Foundation Center for Biosustainability, Technical University of Denmark, 2800 Kgs. Lyngby, Denmark

^2^ Department of Biotechnology and Biomedicine, Technical University of Denmark, 2800 Kgs. Lyngby, Denmark

^3^ Novonesis, 2800 Kongens, Lyngby, Denmark

^4^ InstaDeep Ltd, 5 Merchant Square, London, W2 1AY, UK

^5^ Center for Translational Protein Design, Technical University of Denmark, 2800 Kgs. Lyngby, Denmark

^6^ Department of Bionanoscience, Delft University of Technology, 2629 HZ Delft, Netherlands

^7^ Kavli Institute of Nanoscience, 2629 HZ Delft, Netherlands

*to whom correspondence should be addressed: Alberto Santos ([albsad@biosustain.dtu.dk](mailto:albsad@biosustain.dtu.dk)), Konstantinos Kalogeropoulos ([konka@dtu.dk](mailto:konka@dtu.dk))

**Supplementary Figure Captions**

**Supplementary** **Figure 1. Confidence score distributions across proteases in BSA.** Kernel density estimates of peptide confidence scores for ten proteases, displayed on a logarithmic (base 10) scale. Each curve represents the distribution of predicted peptide confidence for one protease. Distributions are vertically offset and scaled to allow direct comparison across proteases.

**Supplementary** **Figure 2. Comparative analysis of BSA coverage using different proteases.** (A) Number of peptide spectrum matches for protein digest and incubation time optimization for the trypsin protease. A total amount of 50 or 100 µg of BSA was digested for 1 hour, 4 hours, or overnight. (B) Number of peptide spectrum matches for incubation time optimization for each condition tested with chymotrypsin (n=3, error bar showing standard deviation). (C) Pareto plot showing the incremental scaffold coverage gain (Δ coverage) ranked in descending order by protease. Chymotrypsin accounts for the largest gain, followed by substantial contributions from Elastase, Trypsin, and Legumain, while the remaining proteases provide only minor increases. The dotted line indicates the cumulative percentage of coverage gain. (D) Leave-one-out analysis, displaying the scaffold coverage retained when specific proteases are excluded from the set. Excluding trypsin or legumain results in the lowest retained coverage, confirming their major contribution to overall performance. (E) UpSet plot illustrating contig coverage achieved by combinations of the four top-performing proteases (trypsin, elastase, chymotrypsin, legumain). Orange bars represent coverage values for each protease combination, while the connected dots below indicate the specific proteases included. The highest coverage is achieved when all four proteases are combined.

**Supplementary Figure 3. Heatmap of grid search on BSA for DBG scaffold assembly.** Effect of varying assembly parameters on the composite score in graph-based assembly of BSA. Each row represents a combination of k-mer size (k) and minimum overlap (mo), while columns represent size threshold (st) and FDR threshold (fdr). Composite scores are color-coded, with darker red indicating higher values. Higher scores are observed particularly at higher FDR thresholds (0.1 and 0.2) and moderate-high k-mer size (6 and 7). High size thresholds (st = 10) combined with higher FDR values tend to yield the most robust assembly results.

**Supplementary Figure 4. Coverage comparison in nanobody assemblies and contig mapping to a representative nanobody.** (A) Sequence coverage for ten nanobody assemblies (Nb1–Nb10), comparing contig (light blue) and scaffold (dark blue) coverage. High coverage was achieved across all samples; scaffolds generally showed equal or improved coverage compared to contigs, with the exception of nanobody 4. (B) Mapping of contigs to the reference sequence for nanobody 6. Blue bars represent aligned contigs, with red and black markers indicating mismatches and substitutions. The alignment shows high accuracy, with only a single sequence containing a mismatch and another containing a deamidation event, both located in the central region.

**Supplementary Figure 5. Comparison of monoclonal and oligoclonal antibody sequencing metrics for monoclonal antibody 2 and 3, heavy and light chain.** (A) Radar plots showing scaled sequencing metrics for heavy chain (left) and light chain (right) for antibody 2. For the heavy chain, mean identity, coverage, N50, and number of scaffolds are comparable between conditions, except for maximum length, which is superior in the monoclonal sample. In the light chain, the monoclonal sample outperforms the mixture across all metrics, with the exception of mean identity. (B) Comparison for antibody 3. In the heavy chain, the monoclonal sample yields a higher number of scaffolds, while coverage performs slightly better in the oligoclonal mixture; other metrics remain largely similar. Regarding the light chain, mean identity and number of scaffolds are comparable, whereas the monoclonal sample exhibits higher values for the remaining metrics (N50, maximum length, and coverage).

**Supplementary Figure 6. Impact of protein input amount on monoclonal antibody 1 assembly coverage. (A)** Comparison of PSM coverage between low (2 µg, red) and high (2 µg, green) protein starting input amounts. High sequence coverage (>95%) is observed for both light and heavy chains regardless of the starting material, with the high input condition showing only a marginal advantage in the light chain. (B) Evaluation of final scaffold assembly coverage. The light chain assembly demonstrates sensitivity to input amount, showing improved coverage in the high input (89.7%) compared to the low input sample (76.2%). The heavy chain maintains robust assembly performance even with low input, surprisingly yielding slightly higher coverage (87.8%) than the high input condition.

**Supplementary** **Figure 7. Aggregating assembly performance across protein categories using DBG algorithm.** Sequence coverage percentages for different FDR thresholds (1%, 5%, 10%, 20%, 40% and 100%) are plotted for each category. The solid lines represent the mean sequence coverage across all biological samples that belong to the same category, while the translucent shaded bands indicate the 95% confidence intervals which highlights the variability in assembly performance.

**Supplementary Figure 8. Comparative assembly performance of InstaNexus and ALPS across in the samples used in this study, across sequence coverage, longest sequence yielded, and scaffold accuracy. (A)** Sequence coverage plotted against FDR thresholds. ALPS (red) generally yields higher coverage across datasets, particularly at the strictest 1% FDR threshold. However, InstaNexus (blue) achieves comparable coverage to ALPS at the 10% FDR threshold for BSA and Nanobodies, while ALPS maintains a clear advantage for minibinders. (B) Analysis of the longest sequence reconstructed with high identity (>95%). ALPS generates longer sequences, most notably for BSA and antibodies across all FDR thresholds, while nanobodies show competitive lengths at the 5% threshold. (C) Assessment of assembly accuracy. InstaNexus consistently outperforms ALPS in sequence accuracy across all datasets and FDR thresholds, maintaining exceptionally high mean identity values, particularly at the 1% threshold where ALPS shows reduced accuracy.

**Supplementary Figure 9.** **Comparative assembly performance of InstaNexus and ALPS across in the samples used in this study, across N50, sequences mapped to the reference sequence, and total scaffolds produced.** (A) Assembly contiguity evaluated using the N50 metric. InstaNexus (blue) demonstrates superior contiguity compared to ALPS (red), particularly for nanobodies and antibodies, where it consistently achieves higher N50 values, most notably at the 5% FDR threshold. (B) Evaluation of assembly precision (percentage of mapped scaffolds). InstaNexus shows higher efficiency, with a greater proportion of generated sequences mapping to the reference. This advantage is especially evident in nanobodies and antibodies at the stricter 1% FDR threshold. (C) Analysis of fragmentation (total number of assembled sequences produced). ALPS consistently generates a significantly higher number of sequences across all datasets and FDR thresholds, indicating a higher degree of fragmentation compared to the more concise assemblies produced by InstaNexus.

**Supplementary tables**

| Accession code | Category | ID |
| --- | --- | --- |
| TPL0431_01_A07 | Nanobody | Nb1 |
| TPL0604_01_E02 | Nanobody | Nb2 |
| TPL0465_01_G08 | Nanobody | Nb3 |
| TPL0464_01_G08 | Nanobody | Nb4 |
| TPL0611_01_C09 | Nanobody | Nb5 |
| TPL0471_01_B06 | Nanobody | Nb6 |
| TPL0612_01_A09 | Nanobody | Nb7 |
| TPL0604_01_E02 | Nanobody | Nb8 |
| TPL0615_01_E01 | Nanobody | Nb9 |
| TPL0599_01_G01 | Nanobody | Nb10 |
| TPL2555_01_A01 | Antibody | mAb1 |
| TPL0552_02_A05 | Antibody | mAb2 |
| TPL0039_05_A03 | Antibody | mAb3 |
| SILSY1-B11_link2 | Minibinder | miBd1 |
| SILSY1-G05_link2 | Minibinder | miBd2 |
| NY1-B4 | Minibinder | miBd3 |

***Supplementary Table 1. Accession codes for nanobodies, antibodies and minibinders with their IDs in the fasta files used.***

| sample | sequence | # sequences | N50 | coverage | Mean identity | Composite score |
| --- | --- | --- | --- | --- | --- | --- |
| Nb1 | contig | 9 | 22 | 0.91 | 1.00 | 0.84 |
| Nb1 | scaffold | 6 | 30 | 0.91 | 1.00 | 0.95 |
| Nb2 | contig | 9 | 22 | 0.86 | 1.00 | 0.83 |
| Nb2 | scaffold | 6 | 25 | 0.90 | 0.98 | 0.93 |
| Nb3 | contig | 7 | 30 | 0.67 | 1.00 | 0.68 |
| Nb3 | scaffold | 4 | 61 | 0.74 | 0.98 | 0.84 |
| Nb4 | contig | 9 | 25 | 0.94 | 0.98 | 0.82 |
| Nb4 | scaffold | 7 | 31 | 0.94 | 0.97 | 0.89 |
| Nb5 | contig | 9 | 22 | 0.76 | 0.99 | 0.69 |
| Nb5 | scaffold | 5 | 48 | 0.60 | 1.00 | 0.79 |
| Nb6 | contig | 22 | 17 | 0.75 | 0.98 | 0.76 |
| Nb6 | scaffold | 18 | 22 | 0.87 | 0.98 | 0.90 |
| Nb7 | contig | 8 | 27 | 0.91 | 0.98 | 0.90 |
| Nb7 | scaffold | 6 | 29 | 0.91 | 0.98 | 0.92 |
| Nb8 | contig | 13 | 23 | 0.94 | 0.98 | 0.83 |
| Nb8 | scaffold | 10 | 25 | 0.94 | 0.98 | 0.87 |
| Nb9 | contig | 9 | 21 | 0.96 | 1.00 | 0.79 |
| Nb9 | scaffold | 8 | 30 | 0.99 | 1.00 | 0.94 |
| Nb10 | contig | 10 | 21 | 0.96 | 1.00 | 0.82 |
| Nb10 | scaffold | 6 | 32 | 0.97 | 1.00 | 0.97 |

***Supplementary Table 2. Assembly metrics for nanobodies using greedy assembly on scaffolds.*** *Summary of sequence assembly statistics for ten nanobody samples (Nb1–Nb10) processed with the greedy assembly method. Nb10 achieved the highest composite score (0.97), while Nb3 achieved the lowest (0.68).*

| sample | sequence | # sequences | N50 | coverage | Mean identity | Composite score |
| --- | --- | --- | --- | --- | --- | --- |
| mAb1 - H | contig | 41 | 24 | 0.87 | 0.96 | 0.74 |
| mAb1 - H | scaffold | 46 | 44 | 0.89 | 0.99 | 0.86 |
| mAb1 - L | contig | 40 | 28 | 0.95 | 1.00 | 0.88 |
| mAb1 - L | scaffold | 46 | 32 | 0.99 | 1.00 | 0.95 |
| mAb2 - H | contig | 41 | 28 | 0.81 | 1.00 | 0.89 |
| mAb2 - H | scaffold | 41 | 28 | 0.81 | 1.00 | 0.89 |
| mAb2 - L | contig | 48 | 29 | 0.97 | 0.98 | 0.73 |
| mAb2 - L | scaffold | 38 | 46 | 0.89 | 1.00 | 0.86 |
| mAb3 - H | contig | 38 | 22 | 0.83 | 1.00 | 0.93 |
| mAb3 - H | scaffold | 38 | 22 | 0.83 | 1.00 | 0.93 |
| mAb3 - L | contig | 37 | 20 | 0.95 | 1.00 | 0.71 |
| mAb3 - L | scaffold | 38 | 34 | 0.91 | 1.00 | 0.73 |

***Supplementary Table 3. Assembly metrics for mAbs using DBG assembly on contigs and scaffolds.*** *Sequence assembly statistics for three mAbs (mAb1, mAb2 and mAb3), each with light and heavy chains assembled using the DBG method. The mAb1 light chain showed the best performance, achieving a composite score of 0.95 (46 scaffolds, N50 of 32 and a mean identity of 1). For heavy chains, the top result was obtained for mAb 3, with a composite score of 0.93.*

| sample | sequence | # sequences | N50 | coverage | Mean identity | Composite score |
| --- | --- | --- | --- | --- | --- | --- |
| miBd1 | contig | 10 | 15 | 0.49 | 0.99 | 0.64 |
| miBd1 | scaffold | 9 | 26 | 0.53 | 0.99 | 0.81 |
| miBd2 | contig | 11 | 13 | 0.50 | 0.98 | 0.64 |
| miBd2 | scaffold | 7 | 28 | 0.32 | 0.94 | 0.65 |
| miBd3 | contig | 36 | 20 | 0.58 | 1.00 | 0.84 |
| miBd3 | scaffold | 33 | 20 | 0.43 | 1.00 | 0.77 |

***Supplementary Table 4. Assembly metrics for miBds using DBG assembly on contigs and scaffolds.*** *The table summarizes the statistics for three miBds. The best-performing binder was miBd3, achieving the highest composite score (0.84 for contigs), supported by 36 sequences, an N50 of 20, and a coverage of 0.58.*

| Category | FDR | Size threshold | Minimum weight | K-mer size | refinement |
| --- | --- | --- | --- | --- | --- |
| BSA | 0.20 | 10 | 2 | 6 | False |
| Nanobodies | 0.20 | 10 | 3 | 6 | False |
| Antibodies | 0.20 | 10 | 3 | 6 | False |
| Minibinders | 0.20 | 10 | 3 | 7 | False |

***Supplementary Table 5. Mode values of DBG assembly parameters observed across different protein categories.***

*Mode values of key parameters used during DBG scaffold-based assembly for four protein types: BSA, Nbs, miBds, and mAbs. Most parameters show high consistency across categories: FDR (0.20), size threshold (10), and refinement (False) were identical for all groups. The minimum weight was 3 for all categories except BSA (2), while the k-mer size was 6 for all except Minibinders (7).*

| Category | FDR | Size threshold | Minimum overlap | Max mismatches | refinement |
| --- | --- | --- | --- | --- | --- |
| BSA | 0.20 | 10 | 4 | 10 | False |
| Nanobodies | 0.10 | 10 | 3 | 10 | False |
| Antibodies | 0.10 | 10 | 4 | 10 | False |
| Minibinders | 0.1 | 0 | 4 | 10 | False |

***Supplementary Table 6. Mode values of greedy assembly parameters observed across different protein categories.*** *Mode values of key parameters used during greedy scaffold-based assembly for four protein types: BSA, Nbs, miBds, and mAbs. Most parameters are consistent across categories, with minimum overlap fixed at 3 in all cases, and identical confidence (0.88) observed for three out of four categories. Size threshold and minimum identity also show conserved values in most categories, with slight variation for miBds.*
